# WUSCHEL Transcription Factor: From Stem Cell Maintenance to Crop Improvement

**DOI:** 10.1002/advs.202519705

**Published:** 2026-03-31

**Authors:** Zishan Ahmad, Muthusamy Ramakrishnan, Rajeev K. Varshney, Anwar Shahzad, Shamsur Rehman, Bijaya Pant, Qiang Wei

**Affiliations:** ^1^ State Key Laboratory of Tree Genetics and Breeding Co‐Innovation Center for Sustainable Forestry in Southern China Bamboo Research Institute Nanjing Forestry University Nanjing Jiangsu China; ^2^ WA State Agricultural Biotechnology Centre Centre for Crop and Food Innovation Food Futures Institute Murdoch University Murdoch WA Australia; ^3^ Plant Biotechnology Section Department of Botany Aligarh Muslim University Aligarh India; ^4^ State Key Laboratory of Wheat Improvement Peking University Institute of Advanced Agricultural Sciences Shandong Laboratory of Advanced Agriculture Sciences in Weifang Weifang Shandong China; ^5^ Central Department of Botany Tribhuvan University Kirtipur Nepal

**Keywords:** CRISPR/Cas, crop biotechnology, genetic engineering, regeneration competence, WUSCHEL

## Abstract

The WUSCHEL (WUS) transcription factor, long recognized as a master regulator of stem cell maintenance in the shoot apical meristem (SAM), has expanded in significance as a multifaceted tool in plant biotechnology. With an emphasis on its new uses in crop regeneration, somatic embryogenesis (SE), stress tolerance, and developmental regulation in cereals, legumes, and other plant species, this review summarizes recent developments on WUS function outside of *Arabidopsis*. We emphasize how insights from WUS biology can be translated into practical strategies to improve yield, adaptability, and resilience, while also enhancing in vitro tissue culture systems. The objective of this review is to establish WUS as a crucial molecular target for future crop genetic improvement and sustainable farming methods by highlighting the current knowledge gaps and suggesting future directions.

## Graphical abstract

This review synthesizes emerging insights into WUSCHEL (WUS) as a central regulator of plant stem cell fate beyond *Arabidopsis*, highlighting its roles in regeneration, somatic embryogenesis (SE), and stress adaptation across crops. It explores how WUS‐centered regulatory networks, genome editing, and AI‐guided strategies can be leveraged for precise, next‐generation crop improvement.

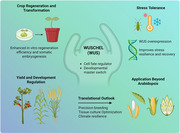

## 1 Introduction

​WUSCHEL (*WUS*) has been pivotal in the field of plant biology as a transcriptional regulator influencing stem cell fate and meristem development, especially in *Arabidopsis thaliana* [1]. It belongs to WUSCHEL‐related homeobox (WOX) gene family, first identified in *Arabidopsis* and essential for the regulation of stem cell identity, ensuring the continuous growth and development of plant part [2]. *WUS* maintains the undifferentiated stem cell pool by controlling the expression of key genes such as CLAVATA3 (*CLV3*) and members of the SPL family, forming a feedback loop that balances stem cell proliferation and differentiation [3]. *WUS* has become a key player in a variety of developmental processes, including reproductive development, organ size regulation, somatic embryogenesis (SE), vascular organization, and stress responses, in addition to its traditional function in stem cell maintenance [4, 5]. Its broad functionality makes *WUS* a promising target for improving plant growth, productivity, and resilience. In crops, *WUS* orthologs influence spikelet formation, grain development, and meristem activity in species such as rice, wheat, maize, and tomato, directly affecting yield, fruit size, and kernel weight [2, 6, 7]. Additionally, the CLV‐WUS regulatory pathway interacts with quantitative trait loci (QTL) that govern agronomic traits such as fruit set and tiller number, giving breeders specific targets to work with [8, 9].

**FIGURE 1:**
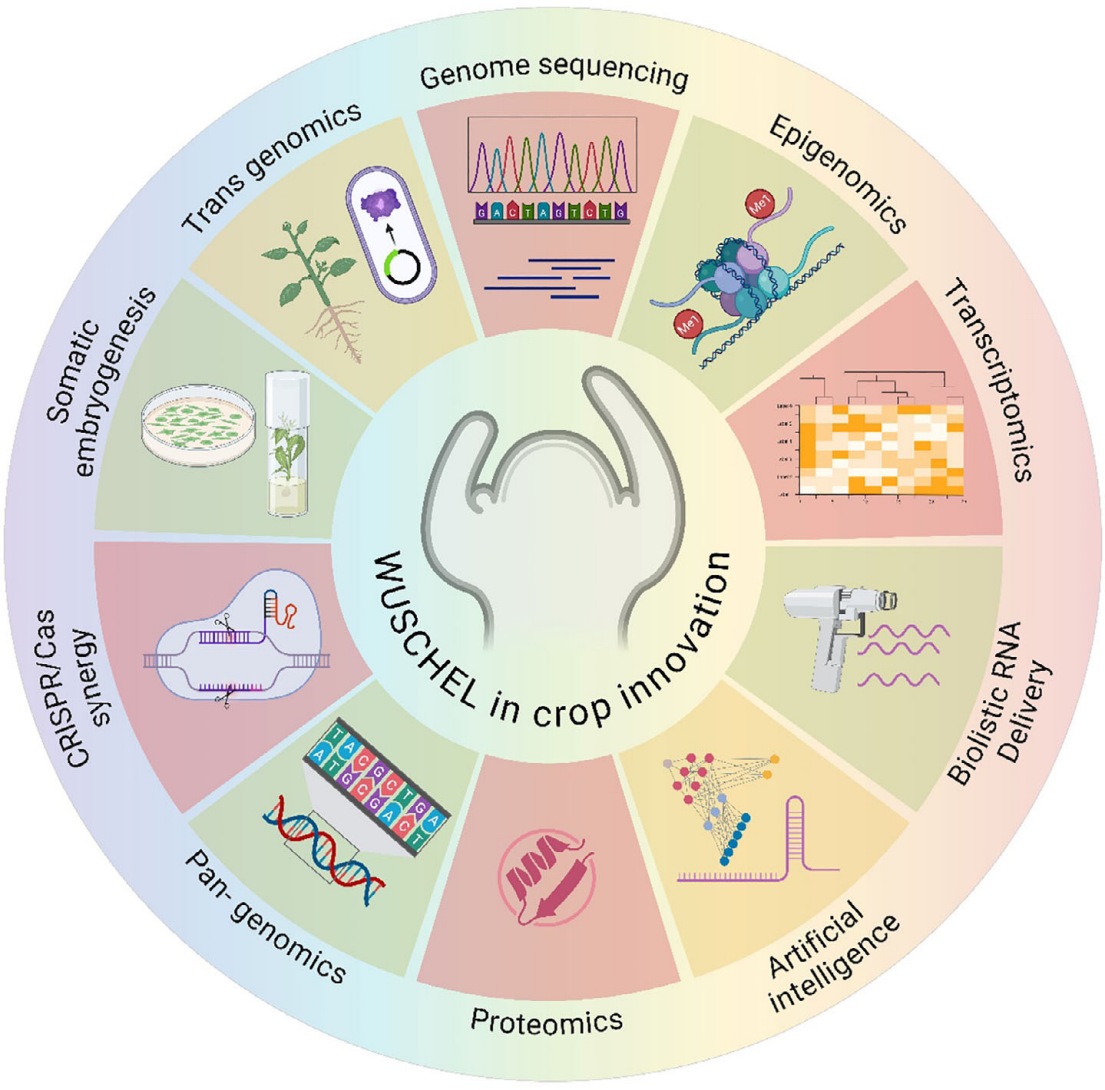
**A conceptual framework demonstrating WUSCHEL's (*WUS*) diverse contribution to crop innovation**. *WUS*, originally discovered as a key regulator of stem cell maintenance in *Arabidopsis*, is now recognized as a central hub for crop improvement. The translational potential of *WUS* manipulation for improving crop yield, organ development, tissue regeneration, and stress responses is increased by developments in biotechnology (CRISPR‐Cas, RNA delivery, trans genomics, somatic embryogenesis (SE)), artificial intelligence, and multi‐omics (genomics, transcriptomics, proteomics, epigenomics, and pan‐genomics).

Recent technological advances including multi‐omics, epigenomics, synthetic biology, and CRISPR‐Cas genome editing have opened unprecedented avenues to manipulate *WUS* for improving plant regeneration, stress tolerance, and productivity. Accordingly, multi‐omics techniques enable a thorough, systems‐level understanding of *WUS*‐mediated control in agricultural species. Identification of *WUS* orthologs, evaluation of allelic variation, and assessment of evolutionary conservation across many taxa are all made possible by genomic and pan‐genomic studies. At the same time, transcriptome analyses clarify the expression patterns of *WUS*‐regulated networks that are particular to cell types, developmental, and stress‐responsive. Additionally, the posttranslational regulation and chromatin‐mediated control of *WUS* function are clarified by proteomic and epigenomic study. These disparate datasets are integrated to enable breeding and genome‐editing efforts aimed at improving yield‐related traits, stress tolerance, and regeneration capability (Figure 1). Mechanistic understanding that can be applied to biotechnological applications is provided by insights into *WUS* regulation through *CLV* signaling, auxin‐mediated crosstalk, and epigenetic modification. Furthermore, *WUS’*s function in SE and tissue regeneration often in conjunction with other members of the WOX family highlights its potential to improve crop genetics and transformation efficiency.

In this review, our aim is to provide an updated synthesis of *WUS* functionality, focusing on its expanding roles beyond stem cell maintenance, including reproductive development, organ size regulation, and stress response. We will explore how recent advances in *WUS* research across various crops, such as wheat, rice, and maize, contribute to crop improvement strategies. Additionally, we will discuss the potential applications of *WUS* manipulation using gene‐editing tools such as CRISPR‐Cas systems for enhancing yield, biomass, and other critical agronomic traits in crop breeding programs.

## 2 *WUS* in Meristem Maintenance

**FIGURE 2:**
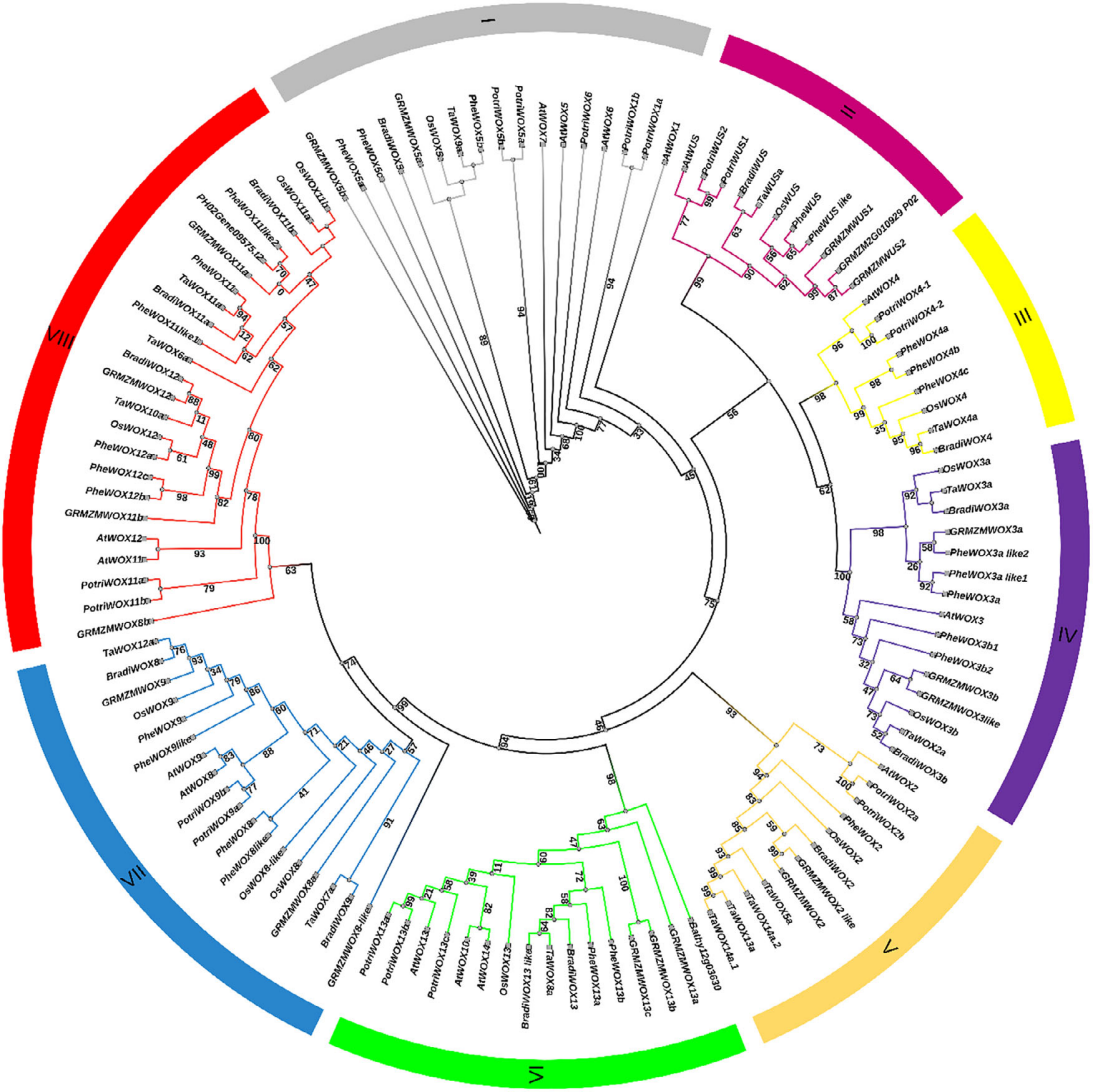
Phylogenetic tree analysis of WUSCHEL‐RELATED HOMEOBOX (WOX) proteins from *Arabidopsis, Triticum aestivum* L*., Bathycoccus prasinos, Brachypodium distachyon, Zea mays, Oryza sativa subsp. Japonica, Phyllostachys edulis, Populus trichocarpa*, was performed using sequences from TAIR10, Ensembl Plants, ORCAE, JGI, MaizeSequence, MSU, and Genome databases. The phylogenetic tree was constructed using the Maximum Likelihood algorithm using the TBTOOLS software and then visualized through (Interactive Tree of Life) (IToL) v. 4 (https://itol.embl.de/), scale bars correspond to 0.1 substitution. The *WOX* genes were resolved into eight distinct clades, which can be broadly categorized into three major evolutionary lineages (ancient, intermediate, and WUS/modern). The number in the middle of each branch represents the bootstrap value (the bootstrap value is presented within a range of 0 to 100).

The foundation for regeneration in both plants and animals is made up of stem cells, a special kind of cell with the capacity to differentiate into numerous cell types [10]. The lifespan and ability to stimulate postembryonic growth of plant stem cells, which are found in meristems, set them apart from animal stem cells. The ability of numerous differentiated cell types to de‐differentiate is another amazing aspect of plant developmental pathway [11] In the shoot apical meristem (SAM), *WUS*, a crucial member of the WOX gene family, is essential for preserving stem cell identity and guaranteeing the ongoing growth and development of plant organs. This gene family, which is highly conserved across a variety of plant species, was initially discovered in *Arabidopsis*. According to comparative phylogenetic analysis of WOX proteins from monocots, dicots and green algae revealed that the WOX family is resolved into eight well supported clades (Figure 2). These clades reflect the evolutionary diversification of *WOX* genes across plant lineage. These eight clades, which reflect different stages of functional specialization during plant development, can be roughly categorized into three major evolutionary lineages: ancient, intermediate, and *WUS* (modern), in accordance with earlier classifications [12, 13]. The *WUS*/modern lineage includes *WUS* and closely related homologs that are functionally specialized in stem cell maintenance and meristem regulation in angiosperms, while the ancient lineage consists of basal *WOX* members linked to early developmental processes and the intermediate lineage represents transitional forms with roles in organ development. Therefore, subclade diversity within these three broad evolutionary lineages is depicted by the eight clades in Figure 2. Further structural analysis of WUS/WOX proteins reveals the presence of conserved homeodomain motifs and unique lineage‐specific features (Figures S1 and S2). These motifs are crucial for DNA‐binding specificity and transcriptional regulation during meristem development and organogenesis. The domain architecture reflects functional conservation among orthologs and offers insights into how structural variation might contribute to distinct regulatory roles in different species.

**FIGURE 3:**
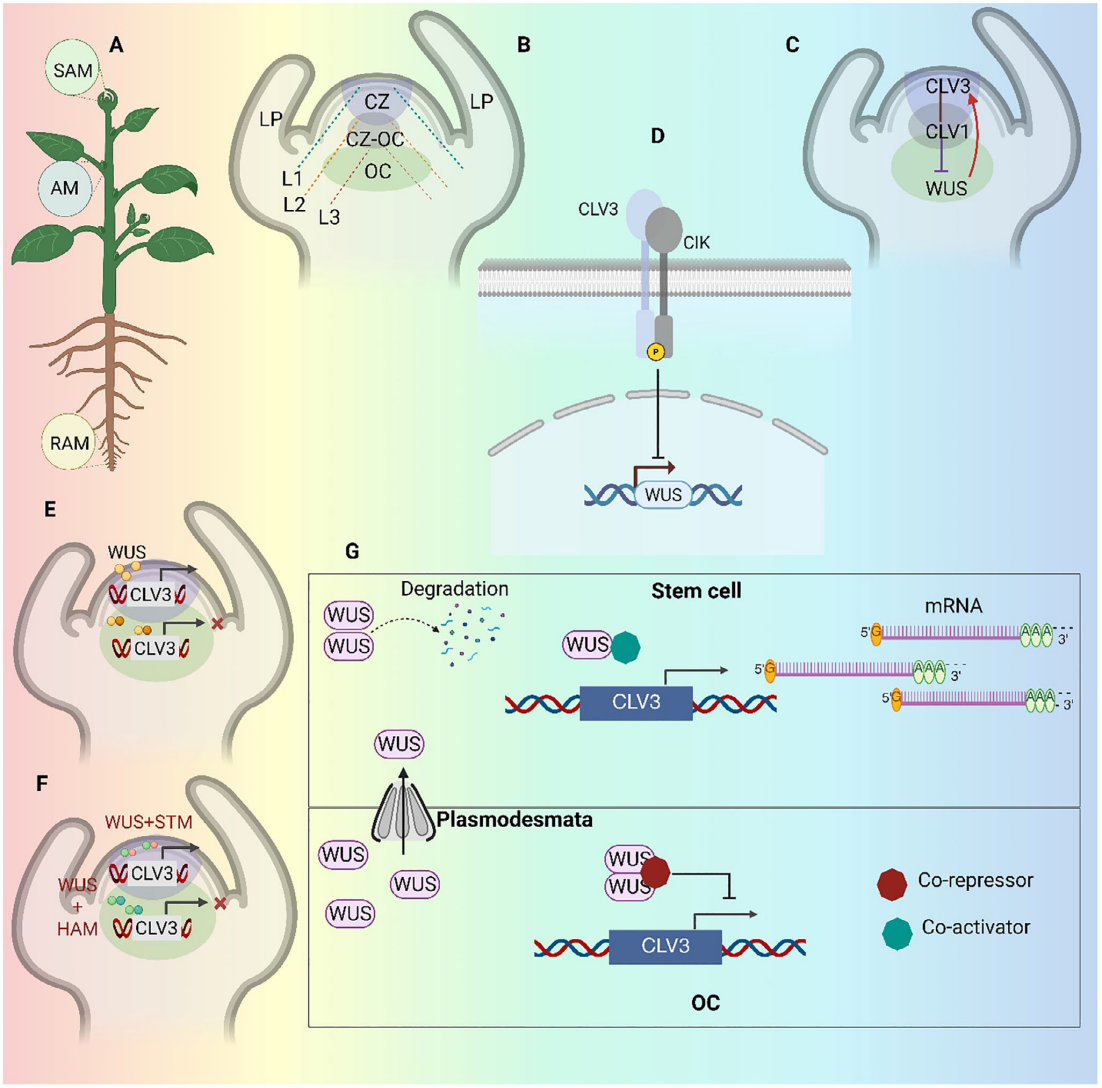
**Meristem organization and CLAVATA‐WUSCHEL (CLV–WUS) regulatory framework**. (**A)** Relative positions of root apical meristem (RAM), shoot apical meristem (SAM), and axillary meristems (AM) in plant, highlighting their hierarchical organization. Only the SAM is detailed in subsequent panels. (**B)** Structural organization of the *Arabidopsis* SAM showing the central zone (CZ), organizing center (OC), cell layers, and emerging leaf primordia. (**C)** Core CLV–WUS negative feedback loop maintaining stem cell homeostasis. (**D)** CLV3 perception via the CLV1–CIK receptor complex and subsequent repression of *WUS* transcription. (**E)** *WUS* dosage‐dependent regulation of *CLV3* expression. In the organizing center (OC), elevated WUS levels facilitate dimerization, which interacts with co‐repressors to inhibit *CLV3* transcription. As WUS permeates the CZ, its concentration diminishes, facilitating monomeric binding to *CLV3* regulatory elements in conjunction with co‐activators, thus enhancing *CLV3* expression. This concentration‐dependent mechanism elucidates the spatial segregation between *WUS* and *CLV3* expression zones and guarantees accurate feedback regulation inside the SAM. (**F)** WUS–HAM interactions refine *CLV3* repression in the organizing center. In the basal OC layer, WUS forms heterodimers with HAM transcription factors, so augmenting the suppression of *CLV3* and confining its expression to the overlying CZ. Conversely, WUS independently interacts with STM in the CZ to facilitate *CLV3* activation, thus orchestrating domain‐specific transcriptional responses that preserve meristem structure and stem‐cell identity. (**G)** Integrated schematic summarizing WUS dose‐dependent control of *CLV3* expression across SAM domains. Increased WUS levels in the OC promote the production of WUS dimers, which subsequently engage with co‐repressors to inhibit *CLV3* transcription. As WUS traverses plasmodesmata into the CZ, its concentration decreases, facilitating monomeric binding to CLV3 regulatory areas in conjunction with co‐activators, thereby commencing *CLV3* expression. This gradient‐dependent control guarantees the geographical separation of *WUS* and *CLV3* domains. In contrast, when WUS accumulates excessively in stem cells, it becomes unstable and is subsequently marked for destruction, thus preserving ideal WUS levels and preventing inappropriate activation within the SAM.

Plants may adapt their growth in response to abiotic and biotic stimuli due to their unique development flexibility. In plant growth region such as shoot, root, and vascular meristems, stem cells are localized within specialized zones known as “stem cell niches”. In stem cell niches, organizing centers (OCs) act as crucial hubs, sending local signals that define and support the nearby stem cell environment. These signals establish a specialized microenvironment that is essential for maintaining stem cell identity and promoting self‐renewal. By carefully modulating biochemical cues and regulatory factors, OCs ensure that stem cells receive targeted support without triggering premature differentiation [14]. This balance in signaling fosters a delicate cellular equilibrium, crucial for developmental processes and stress adaptation. In essence, OCs serve as both guardians and guides, preserving the potential of stem cells to respond dynamically to various environmental and physiological demands [11, 14]. Primary growth at the shoot apex is driven by SAM (Figure 3A). Although not all plants have this layout, the SAM in many are arranged in three cell layers: epidermal layer (L1), subepidermal layer (L2), and the L3 that will produce vascular and stem tissues. The OC, a collection of cells that support stem cell identity within the SAM, is located inside the L2/L3 layer. An axillary meristem (AM) forms inside each leaf's axil as the stalk grows, and the SAM produces the lateral organ primordia that will eventually become leaves (Figure 3B) [10].

AMs as well as SAMs start off as stem cells that give rise to vegetative structures, such as leaves and branches. When florigen is generated and circulates throughout the plant systemically, floral induction takes place. Florigen acquires the identity of an inflorescence meristem (IM) or floral meristem (FM) as it reaches meristems. These pools of stem cells are key to forming the structured, geometric arrangement of leaves, stems, and roots, giving rise to the organized architecture of the plant [14]. The control of meristem function and maintenance in plants involves several intricate signaling pathways that coordinate stem cell activity and differentiation. In this section, we will describe about the involvement of *WUS* in stem cell maintenance.

### 2.1 CLAVATA‐WUSCHEL (CLV‐WUS) Pathway

The CLV signaling pathways is central to the regulation of the SAM in plants. Continuous plant development and organ formation depend on the CLV‐WUS signaling system in the SAM maintaining a crucial balance between stem cell proliferation and differentiation. The WUS TFs which promotes stem cell identity, is expressed in the OC cells. The WUS protein moves outwards through plasmodesmata to reach the L1 layer cells at the shoot apex [15] (Figure 3C). At the center of this pathway is WUS TFs, activates CLAVATA3 (*CLV3*) expression to promote stem cell identity. To become functional, *CLV3*, a founding member of the CLV3/EMBRYO‐SURROUNDING REGION (CLE) peptide family, must go through post‐translational changes such as cleavage into a short peptide and the addition of arabinosyl sugars [9, 16]. In order to maintain a steady population of self‐renewing stem cells, this modified CLV3 peptide inhibits WUS production by binding to a group of leucine‐rich repeat (LRR) receptor kinases after it is secreted (Figure 3D) [17]. This basic CLV3–WUS feedback loop, which balances stem cell renewal and differentiation needed to produce new organs, is the cornerstone of meristem maintenance, as seen in Figure 3C,D. New CLE peptides and receptor components have been discovered in recent research that goes beyond the basic concept. Together, these elements link CLV signaling to hormone and gene expression regulation, which aids in regulating the activity of the SAM [11, 18]. These results show that the CLV–WUS pathway functions as a dynamic signaling hub that coordinates several developmental inputs rather than as an isolated loop.

In order to maintain a balanced interaction between stem cells and differentiated tissues in the SAM, CLV3/CLE peptides are essential for the transcriptional regulation of *WUS*. Through a feedback loop in which *WUS* stimulates *CLV3* expression and *CLV3* represses *WUS*, this equilibrium is preserved. A significant question is brought up by this mutual regulation: how does *WUS* continue to express itself, particularly at the OC, in spite of *CLV3*’s repressive influence? The spatial specificity of SAM is maintained by HAIRY MERISTEM (HAM) genes, which are expressed in the basal areas of the SAM and block CLV3 activation within the organizing center (OC) [19] (Figure 3E). On the other hand, *WUS* and SHOOT MERISTEMLESS (*STM*) interact in the central zone to promote *CLV3* expression (Figure 3F). Moreover, HAM proteins influence SAM patterning and stem cell identity by creating an apical–basal gradient throughout the meristem. Epidermis‐specific transcription factors, such as ATML1, modify this gradient by controlling HAM levels via microRNA [20]. As a result, WUS activity is spatially constrained by these combined mechanisms, maintaining the ordered structure of the SAM.

Another study revealed that *WUS* regulates *CLV3* levels in SAM through dose‐dependent mechanism involving unique interaction with *cis*‐regulatory elements [21, 22]. Figure 3G demonstrates the essential function of WUS dosage gradients in regulating CLV3 transcription across the SAM regions. Increased WUS levels in the organizing center promote dimerization and subsequent suppression of CLV3; in contrast, reduced levels in the covering stem cells encourage monomeric WUS binding and gene activation. Moreover, excessive WUS in stem cells is subject to targeted degradation, ensuring the spatial limitation of WUS activity and maintaining homeostatic balance within the meristem [21]. This concentration‐dependent control preserves the spatial precision of the feedback system by explaining how WUS is produced in the OC without activating CLV3 in the incorrect location. Greater *WUS* concentrations in the OC cause *CLV3* suppression, creating a spatially accurate feedback loop that is necessary for stem cell homeostasis and preserving the equilibrium between stem cell differentiation and self‐renewal in the SAM. When taken as a whole, these mechanisms show how the CLV–WUS network is dynamic and self‐regulating, going beyond a static feedback concept.

In land plants, the regulation of stem cells is essential for the growth and development of organs. The CLV3 peptide, which is produced by *Arabidopsis* stem cells, blocks *WUS* expression through the CLV1 receptor, creating a reciprocal feedback loop in which *WUS* stimulates *CLV3* expression to preserve stem cell identity. Differentiating cells contain CLE40, which is involved in a second loop. *WUS* is activated by this loop via the BAM1 receptor. *WUS* suppresses *CLE40* concurrently. This procedure aids in striking a balance between stem cell differentiation and renewal [23]. The CLV3–CLV1–WUS and CLE40–BAM1–WUS modules, which are connected, work together to maintain the balance of meristems and their ability to respond to developmental signals. This is achieved through a complex system of feedback regulation.

As evidenced by the moss *Physcomitrium patens*, the CLV pathway controls stem cells outside of flowering plants without the assistance of WOX transcription factors, including WUS [24]. This demonstrates that *CLV* signaling has a long history in stem cell organization that predates its connection to WOX factors. Flowering plants' CLV‐WOX feedback loop is probably an evolutionary invention that improved developmental plasticity. Different ancestral modes of stem cell control are suggested by the WOX‐independent CLV mechanism in mosses. The evolutionary diversification of stem cell regulatory networks can thus be elucidated by investigating CLV‐WOX interactions across lineages. These investigations offer new insights into how plant stem cell niches changed over time to satisfy developmental requirements unique to a given lineage. Therefore, the CLV–WUS module represents its evolutionary adaptation for lineage‐specific developmental flexibility in addition to being an example of a conserved stem‐cell regulatory mechanism.

### 2.2 AUX‐WUS‐CKs Crosstalk

The long‐term self‐renewal and differentiation of stem cells are jointly controlled by the CLV‐WUS signaling pathway, as well as the local levels and spatial distribution of auxin and cytokinins (CKs), indicating a coordinated crosstalk between auxin, WUS, and CK. This connection ensures that the control of gene expression and the distribution of hormones work together, balancing the growth of stem cells with the start of organ development. However, the question about how this crosstalk is translated into genetic changes that sustain meristem function and drive shoot regeneration still need to be explored.

By directly controlling cytokinin response regulators, particularly the *ARABIDOPSIS RESPONSE REGULATOR* genes *ARR5/6/7/15*, it is possible to activate cytokinin‐related signaling. In a positive feedback loop, these type‐B ARRs subsequently increase *WUS* expression, strengthening the CK‐WUS connection that promotes stem cell proliferation [25, 26]. In *Arabidopsis*, WUS functions as a molecular switch, fine‐tuning local auxin concentrations within the CZ by modulating *AUXIN RESPONSE FACTORs* (*ARFs*) and influencing chromatin state through histone acetylation. Crucially, data shows that WUS is an integrator of auxin and cytokinin signals, where hormonal crosstalk and epigenetic regulation meet.

**FIGURE 4:**
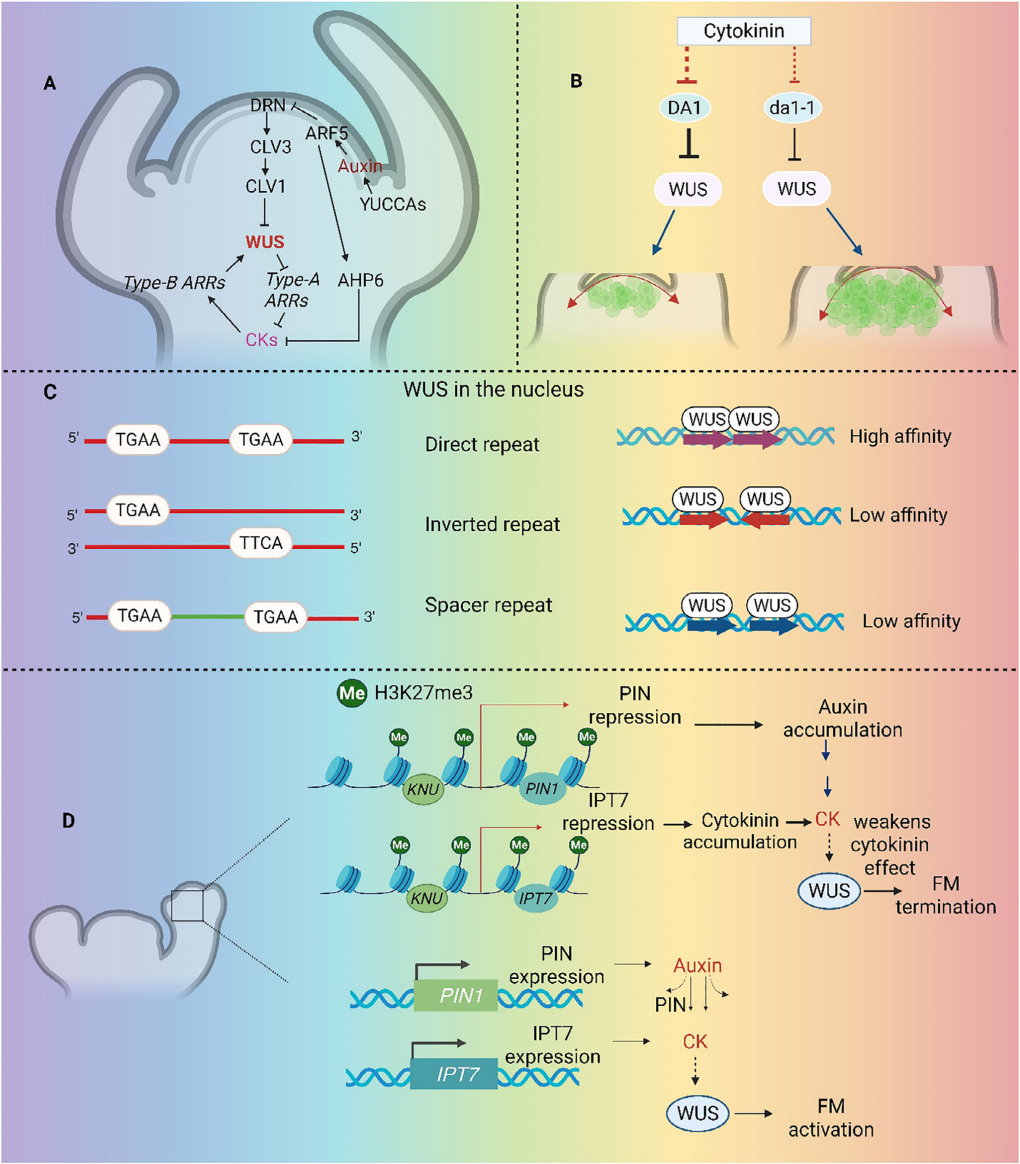
**Integrated regulatory networks of *WUS* activity**. (**A)** Crosstalk between auxin and cytokinin signaling pathways and their interaction with WUSCHEL (WUS). (**B)** Cytokinin–DA1–WUS regulatory module influencing shoot apical meristem (SAM) size and stem cell maintenance. (**C)** DNA motif architecture influencing *WUS* homeodomain binding and dimerization efficiency. (**D)** KNUCKLES‐mediated epigenetic regulation contributing to floral meristem (FM) termination through modulation of WUS activity.

Leibfried et al. [27] established the first connection between WUS and cytokinin signaling by demonstrating that *WUS* directly suppresses the transcription of many *ARABIDOPSIS RESPONSE REGULATOR* (ARR) genes that react to cytokinins, namely *ARR5*, *ARR6*, *ARR7*, and *ARR15*. Because these type‐A *ARR*s are negative regulators in the cytokinin feedback loop, *WUS*’s suppression of these genes is essential for preserving stable cytokinin activity and suitable meristem size. This finding provided the first molecular evidence that *WUS* directly bridges hormonal feedback to stem‐cell maintenance. *ARR7* constitutive activation mutations cause aberrant meristem architecture, whereas larger meristems are linked to loss‐of‐function alleles in maize ARR homologues. Together, these results highlight how *WUS* regulates cytokinin sensitivity to maintain meristem homeostasis [28, 29] (Figure 4A).

Auxin signaling activates *WUS* expression via *ARF5/MONOPTEROS* and *DRN/ESR1*, influencing *CLV3*, while Cytokinin enhance *WUS* through type‐B *ARRs* (*ARR1*/*10/12*) [25, 29]. In *Arabidopsis*, the cytokinin signaling inhibitor *ARABIDOPSIS HISTIDINE PHOSPHOTRANSFER PROTEIN 6* (*AHP6*) is transcriptionally activated by *MONOPTEROS (MP)/ARF5* [30, 31]. In order to preserve the spatial separation between auxin‐ and cytokinin‐rich domains in the SAM, AHP6 migrates from the peripheral zone towards the central stem cell region and locally inhibits CK signaling at organ initiation boundaries. This equilibrium is essential because lateral organ formation is suppressed by a lower CK‐to‐auxin ratio while it is encouraged by a higher one. These pathways jointly demonstrate that WUS is located at the meeting point of two antagonistic hormonal circuits that together shape the SAM.

In a recent study, the peptidase DA1 cleaves the WUS protein, destabilizing it and affecting the control of stem cells in plant shoot meristems. It was discovered that cytokinin signaling suppresses DA1 levels, which increases WUS stability and accumulation [31] (Figure 4B). Cytokinin‐induced meristem expansion is enhanced when DA1 activity is lost because larger SAM with more stem cells is produced. These results uncover a new mechanism by which cytokinin suppresses *DA1* to stabilize WUS, hence encouraging meristem development and stem cell population increase. This discovery integrates hormonal signaling and protein stability by adding a posttranslational regulatory layer to WUS regulation.

Conversely, *WUS* allows stem cells to endure auxin‐induced differentiation while yet needing minimal levels of auxin to sustain themselves. *WUS* does this by adjusting the auxin signaling pathway, which permits stem cell self‐renewal and differentiation signals to coexist in harmony [28]. This dual regulatory mechanism highlights *WUS*’s role as a dynamic coordinator. It balances chromatin states and hormonal signals, rather than simply acting as a downstream effector. In addition, dual regulation of *WUS* was identified in *Arabisopsis*, Type‐B ARRs promote *WUS* expression, crucial for stem cell maintenance, and simultaneously repress auxin accumulation by inhibiting *YUCCA* genes, which are involved in auxin biosynthesis [29]. Together, the *WUS* and ARR modules can improve cytokinin signaling and decrease auxin synthesis because to this feedback mechanism. This technique aids in preserving a steady hormonal balance within the SAM, both in terms of the location and quantity of hormones.

In order to maintain steady gene expression, *WUS* controls histone acetylation at particular target genes, including those implicated in auxin signaling. In the face of fluctuating developmental cues, this system permits accurate and reliable regulation of stem cell behavior. Furthermore, *WUS* refines stem cell identity by increasing histone deacetylation, which suppresses particular target genes within the stem cell domain and controls gene expression at an epigenetic level [32, 33]. Thus, *WUS* functions as a crucial integrator, linking transcriptional, epigenetic, and hormonal processes to preserve stem‐cell identity and regenerative capacity. These observations collectively position *WUS* as a regulatory center, bridging auxin and cytokinin interactions with chromatin‐based transcriptional regulation within the SAM.

### 2.3 Epigenetic Modulation of WUSCHEL Transcription

Plants possess remarkable developmental plasticity that allows them to adjust growth and differentiation in response to both developmental and environmental cues. This adaptability is primarily regulated by epigenetic mechanisms, which influence gene expression without modifying the DNA sequence itself. While much is known about SAM functions such as stem cell maintenance, organ initiation, the molecular insights into how epigenomic mechanisms specifically control the expression of central regulators such as *WUS* are still emerging [34, 35].

#### 2.3.1 Epigenetic Regulation of WUSCHEL Transcription

Through chromatin architecture, histone changes, and DNA methylation, epigenetic regulation at the WUS locus controls transcriptional accessibility without the use of hormone signals. The transcriptional activity or suppression of *WUS* during development and regeneration is determined by changes in chromatin states. To maintain proper *WUS* expression dynamics and guarantee a stable stem cell niche, repressive H3K27me3 marks must be eliminated and activating H3K4me3 marks must be enriched (Watson et al., 2016; You et al., 2017). Additionally, chromatin shape is essential for accurate transcriptional control. AGAMOUS (AG) and TERMINAL FLOWER 2 (*TFL2*/*LHP1*) form a chromatin loop in *Arabidopsis* that connects areas close to the *WUS* gene. This connection suppresses transcription by preventing the recruitment of RNA polymerase II (Guo et al., 2018). During FM determination, this higher order chromatin structure silences WUS by acting as a spatial switch. This demonstrates that one important epigenetic regulatory mechanism is the three‐dimensional structure of the genome (Guo et al., 2018).

The stability of *WUS* transcriptional states is further strengthened by DNA methylation. Due to altered methylation and histone modification patterns within its regulatory areas, *WUS* expression and regeneration efficiency are altered in *met1*, *kyp*, *jmj14*, and *hac1* mutants [36]. These findings highlight the connection between hormone pathways and chromatin dynamics during organogenesis, as methylation not only controls *WUS* expression but also interacts with auxin signaling through the misexpression of *ARF3*. Structural and biochemical studies have enabled a mechanistic understanding of how *WUS* binds and interprets these epigenetic landscapes. *WUS* has a high affinity for TGAA direct repeats and produces dimers via specific homeodomain residues that stabilize DNA binding [37] (Figure 4C). This motif‐dependent binding mechanism explains the context‐dependent regulatory functions of *WUS*, which either activate or repress its targets depending on chromatin conformation and sequence arrangement. Additional proof that chromatin modification plays a key role in WUS‐mediated activation of its target genes was recently presented by a mechanistic study. *WUS* recruits the *SWI/SNF* chromatin‐remodeling ATPase BRAHMA (BRM) to the *CLV3* promoter within the *Arabidopsis* SAM by forming an association with the transcriptional cofactor DORNRÖSCHEN (DRN) [38]. Following nucleosome displacement and RNA polymerase II recruitment, this WUS–DRN–BRM complex activates *CLV3* expression while maintaining stem cell identity. This finding demonstrates that *WUS* not only reacts to epigenetic changes but also actively modifies them to control meristem function, establishing a clear mechanistic link between chromatin remodeling and *WUS*‐mediated transcriptional activation.

#### 2.3.2 Epigenetic Control of WUSCHEL Downstream Targets

*WUS* modulates stem cell activity by influencing the epigenetic environment of its target genes in addition to its direct regulatory actions. Polycomb Repressive Complex 2 (PRC2) is recruited by the transcriptional repressor KNUCKLES (KNU), which functions as an epigenetic antagonist to WUS. This complex then adds H3K27me3 marks to important hormone regulators, such as IPT7 and PIN1. Thus, by efficiently suppressing auxin and cytokinin production pathways, this alteration leads to the termination of the FM and the accurate timing of growth [39] (Figure 4D). On the other hand, this suppression is weakened in the absence of KNU, resulting in extended meristem activity and continuous synthesis of both *WUS* and *CLV3*.

Furthermore, in addition to chromatin remodeling and interactions with transcriptional cofactors that impact WUS's downstream targets, the activity of *WUS* is also connected to environmental and evolutionary factors through epigenetic signaling pathways. One relevant example is the nitric oxide (NO) mediated redox control of the RNA‐directed DNA methylation (RdDM) pathway. NO signaling connects environmental inputs to the epigenetic balance of stem cells by modifying *ARGONAUTE* 4 (*AGO4*), a critical component of RdDM. A direct, NO‐dependent physical connection between *WUS* and AGO4 facilitates this regulatory process, which maintains the integrity of the shoot meristem and refines DNA methylation at genomic sites specific to stem cells [40]. Beyond immediate environmental control, evolutionary forces have also shaped *WUS*‐dependent networks via transposable element (TE) derived regulatory sequences. New *cis*‐regulatory modules are created by TE insertions within the promoters of crucial meristematic genes as *WUS*, *CLV3*, and *STM*. Developmental plasticity and transcriptional responsiveness are improved by these modules [41]. On the other hand, TE activity is suppressed by heterochromatinization and small‐RNA‐mediated silencing, protecting genomic stability but also permitting evolutionary innovation [42, 43].

Together, these findings show that *WUS* uses epigenetic mechanisms to regulate gene expression, uphold repression, and maintain genomic stability at the intersection of transcriptional, chromatin, and environmental regulation. Plant growth and adaptation benefit from this complex epigenetic structure, which ensures that *WUS*‐driven stem cell networks are both flexible and developmentally robust.

## 3 WUS Beyond the Meristem

*WUS* has long been known for its critical function in preserving stem cell populations in the SAM, where it maintains meristematic activity and regulates cell division, both of which are necessary for plant growth. However, recent studies indicate that WUS functions extend beyond meristematic maintenance, significantly impacting plant growth, blooming, and even SE, reflecting a more dynamic regulatory role across developmental stages and environmental conditions. In this section we will discuss the role of WUS and related homeobox gene (WOX) beyond the meristem maintenance.

### 3.1 WUS‐WOX Regulatory Network in Somatic Embryogenesis

SE is the process by which nonreproductive, somatic cells of a plant are reprogrammed to develop into embryos, mimicking the stages of zygotic embryogenesis. Without the need for sexual reproduction, these somatic embryos can later develop into fully functional plants [44]. Advances in genomics and transcriptomics have identified key gene network involved in SE [45]. Different TFs such as SOMATIC EMBRYOGENESIS RECEPTOR‐LIKE KINASE [46], WUSCHEL AND WUSCHEL‐related homeobox (WOX) [47, 48], BABY BOOM (BBM) [49], LEAFY COTYLEDON (LEC) [50], FUSCA (FUS3) [51], AGAMOUS‐LIKE15 (AGL15) [52], WRKY [53, 54], RWP‐RK DOMAIN‐CONTAINING PROTEIN (RKD) [55], auxin response factors (ARFs) [56] and MYB [57] are found to be involved directly or indirectly in regulating the process of SE. Among all TFs, WUS is crucial in SE because it acts as a molecular hub that controls cell fate determination and maintain stem cell pluripotency [2]. The positive regulation of *WUS* and *WOX* in controlling SE has been already reported in different plant species such as *Arabidopsis* [48], *Solanum tuberosum* [58], *Xanthoceras sorbifolia* [59], *Picea abies* [60], *Brachypodium distachyon* [61], and *Medicago sativa* [62]. Table 1 represents the role of *WUS* and *WOX* in SE and plant tissue culture.

**TABLE 1:** *WUS* and *WOX* TFs reported in plants and their role in somatic embryogenesis (SE) and tissue culture.

Plant	Gene	Function	Methods	Phenotypic marker	Remark	References
*Arabidopsis*	*WOX2/5/8/9*	Coordinate embryonic cell fates	Gene expression and mutant analysis	Defective cell derivatives	Apical‐basal polarity establishment	[73]
*Coffea canephora*	*WUS*	Role in SE and morphogenesis	Transgenic expression	Calli formation	Enhanced somatic embryo production	[74]
*Arabidopsis*	*WUS*	SAM maintenance in embryogenesis	Mutant analysis and gene Suppression	Induced somatic embryos	Auxin–WUS–PIN1 axis	[75]
*Picea abies*	*PaWOX2/8/9*	Early and late embryogenesis	In situ hybridization, Treatment with PAT inhibitor	NPA‐induced embryogenesis changes	WOX directs embryonic development	[76]
*Vitis vinifera*	*VvWOX2/9/3/11*	Early somatic embryo regulators	Expression analysis	Embryo formation stages	Culture optimization, genotype selection, stress treatments	[77]
*Ocotea catharinensis*	*OcWUS*	Key for SE and SAM	Transgenic expression	Malformed somatic embryos	Stem cell control in embryogenesis	[78]
*Picea abies*	*PaWOX8/9*	Regulation of embryonic development	RNAi to down regulate the expression	Aberrant embryo morphology	Regulating axis establishment and cell cycle regulation	[60]
*Gossypium hirsutum*	*AtWUS*	Activates auxin‐driven embryogenesis	Ectopic expression of *AtWUS* in cotton cultivar CRI12 using genetic transformation techniques.	*AtWus* boosts EC	overcoming the recalcitrance of cotton somatic embryogenesis (SE)	[63]
*Pinus pinaster*	*PpWOX2*	Role in early embryogenesis	*Agrobacterium* mediated transformation in *Arabidopsis*	non‐embryogenic callus (EC) formation in roots of *P. pinaster*	WOX gene conservation	[79]
*Solanum tuberosum*	*SiWUS*	Stem cell maintenance and differentiation	RNAi to down regulate the expression	WUS enables regeneration	WUS drives regeneration efficiency.	[58]
*Phoebe bournei*	*WOX*	Essential for somatic embryogenesis (SE)	Expression analysis	Callus and embryo development	WOX genes in development	[47]

*WUS* has been identified as a master regulator of SE, with its overexpression enabling embryo formation across diverse tissues without exogenous hormones, revealing a novel intrinsic pathway for regeneration [48]. Later research revealed that *WUS* overcomes constraints in low‐EC cultivars by promoting embryogenic callus (EC) differentiation in cotton by activating important auxin‐related genes such as *LEC1*, *LEC2*, *FUS3*, *PIN7*, and *SHY2* [63]. Complete regeneration was difficult even though *AtWUS* expression enhanced the formation of embryogenic tissue in cotton, indicating the necessity of optimizing phytohormone balance [64]. Cellular research in *Arabidopsis* also showed that *WUS*‐expressing cells mark embryogenic sites, highlighting its role in early embryo induction along with Ca^2+^ fluxes and callose deposition [65]. Tea's conserved function across species was further supported by the discovery that *WUS* and other SE‐related genes were highly expressed during embryo initiation [66]. More recently, the miR394–WUS module was shown to enhance embryogenic potential via repression of LEAF *CURLING RESPONSIVENESS* (*LCR*) gene, revealing an additional epigenetic layer of pluripotency regulation [67].

Genes from the WOX family complement WUS in the processes of embryogenesis and regeneration. *PaWOX8/9* controlled the formation of the apical‐basal axis in *Picea abies*, and RNAi suppression resulted in aberrant embryo polarity and disrupted cell division, underscoring conserved WOX functions in embryonic patterning [60]. *GhWOX11/12* maximized transformation efficiency in *Gossypium hirsutum* by promoting callus initiation, shoot regeneration, and cell fate transitions [68]. Likewise, nine *WOX* genes were identified in *Xanthoceras sorbifolia*, with *XsWOX1*, *4*, and *5* linked to SE and organogenesis via promoter elements that respond to stress and hormones [59]. In *Cunninghamia lanceolata*, *CIWOX5/6* improved shoot regeneration in tobacco, highlighting their cross‐species applicability, while *Wox2a* overexpression in maize allowed SE and regeneration in resistant lines without chemical selection [69].

The *WUS* and *WOX* genes work together to form a conserved regulatory module that controls developmental reprogramming, preserves pluripotency, and initiates embryogenic competence in all plant lineages. Their capacity to overcome genotype‐specific regeneration limitations has significant ramifications for crop biotechnology, facilitating effective tissue culture, genetic transformation, and the creation of stress‐tolerant, high‐yield cultivars. The WUS–WOX network should become a key component of next‐generation crop improvement strategies by concentrating on precise, tissue‐specific, and inducible expression systems that optimize transformation efficiency while reducing pleiotropic effects.

### 3.2 Regulation of Plant Growth and Blooming

WUS, a homeodomain transcription factor, acts as a master regulator of plant development, governing the balance between stem cell maintenance, organ formation, and reproductive transition (Table 2). In tree species such as *Populus trichocarpa*, *WUS* homologs play vital roles in vascular patterning and secondary growth. For example, *PtrWUSa* expression was found to be robust in the vascular cambium and in the developing phloem fibers; furthermore, the repression of *PtrWUSa* in transgenic poplar resulted in disrupted vascular organization, altered stem morphology, and reduced wood production [70]. These observations underscore the conserved function of WUS‐type genes in regulating meristematic activity and tissue differentiation within woody plants, suggesting potential applications in both forestry and bioengineering.

**TABLE 2:** Role of WUSCHEL (WUS) and WUS‐related homeobox (WOX) transcription factors in plant growth and development.

Plant	Gene	Interacting gene	Family	Function	References
*Arabidopsis*	*WUS*	*ANT*	Brassicaceae	Floral and ovule development	[84]
*Solanum lycopersicum*	*SIWUS*	*SICLV3*	Solanaceae	SAM and organ differentiations	[85]
*Glycine max*	*GmWUS*	*CLV*	Fabaceae	Floral initiation	[86]
*Solanum lycopersicum*	*WUS*	*CLV3*	Solanaceae	Development of flower organ identity	[87]
*Oryza sativa*	*WOX4*	*FCP1/FCP2*	Poaceae	*FCP1/2 repress WOX4*	[88]
*Oryza sativa*	*OsWOX13*	*OsDREB1A, OsDREB1F*	Poaceae	Linking stress tolerance and flowering	[89]
*Phalaenopsis equestris*	*PeWOX*	*CLV*	Orchidaceae	Embryo, organ, and flower development	[90]
*Dendrobium catenatum*	*DcWOX*	*CLV*	Orchidaceae	Embryo, organ, and flower development	[90]
*Oryza sativa*	*OsWOX4*	*LOG*	Poaceae	Meristem, vasculature, and leaf initiation	[91]
*Oryza sativa*	*WOX3*	*LSY1*, *NAL2*/*NAL3*	Poaceae	Lateral leaf polarity and development	[92]
*Glycine max*	*GmWOX18*	*NA*	Fabaceae	Stress tolerance and bud regeneration	[93]
*Chrysanthemum morifolium*	*CmWUS*	*CmCYC2d*	Asteraceae	Control of reproductive organogenesis	[5]
*Cucumis melo*	*CmWOX*	Hormonal and stress signaling	Cucurbitaceae	Plant development under abiotic stress	[94]
*Yellowhorn*	*XsWOX1‐5*	Hormonal and stress signaling	Sapindaceae	Role in SE and abiotic responses	[59]
*Cucumis sativus L*.	*CsWOX3*	*CsSPL15*, *CsMIEL1*‐like *CsWOX3*	Cucurbitaceae	Suppresses cucumber fruit spine formation	[4]

*WUS* controls the transition from meristem maintenance to floral induction in addition to vegetative growth. For example, Zhang et al. [71] demonstrated that *WUS* stimulates the development of axillary meristems and branching by activating response regulators (*ARR12*) and cytokinin production‐related genes (*IPT7*). Further investigations revealed that *WUS* interacts with the transcriptional cofactor DORNRÖSCHEN, recruiting the SWI/SNF remodeler BRAHMA to the *CLV3* promoter. This process facilitates chromatin remodeling, which in turn helps maintain meristem size [38]. While *CsLFY*‐ *CsWUS* interaction is required to promote the flower development via activation of CsAP3 and CUM1 in cucumber [72]. In addition, timely repression of *WUS* by KNUCKLES (KNU) is essential for FM termination, ensuring proper determinacy [39].

At the spatial level, a finding explained how intercellular positional signaling limits the expression domains of *WUS* and *CLV3* in the SAM, in addition to these regulatory mechanisms [80]. Together, EPIDERMAL PATTERNING FACTOR LIKE (EPFL) ligands and ERECTA family (ERf) receptor kinases restrict the stem cell domain and promote organ start. EPFL ligands are localized in the periphery of the SAM, where they interact with *CLV3* to modify meristem size, whereas ERf receptors are extensively distributed throughout the SAM. As a result, WUS and *CLV3* expression in the periphery zone is quickly reduced when exogenous EPFLs activate ERf signaling, limiting their action to the central organizing core. Additionally, wus is epistatic to erf mutants, according to genetic investigations, validating ERf–EPFL signaling as an upstream spatial cue that controls stem cell location along the radial axis of the SAM. Across species, *WUS* function is further preserved in inflorescence development and flowering. For example, in loquat (*Eriobotrya japonica*) it was found that that when overexpressed in *Arabidopsis*, *EjWUSa* interacts with *EjSTM* and stimulates early blooming in loquat [81]. Similarly, in maize, *ZmWUS1* was found to be a part of a transcriptional network that regulates ear development and the determination of inflorescence [82].

In their recent single‐cell transcriptome analysis of maize, Sun et al. [83] revealed complex regulatory systems controlling sexual differentiation and meristem identity. The study demonstrated a relationship between the activity of RNA‐binding proteins during the differentiation of axillary and inflorescence meristems and *WUS*‐mediated stem cell control. These results demonstrate the conserved role of *WUS* in regulating hormonal signaling pathways, reproductive development, and stem cell maintenance.

By combining hormone, chromatin, and positional cues, WUS essentially acts as a developmental nexus, coordinating organ creation, meristem identification, and blooming. Its evolutionary conservation from herbaceous to woody plant lineages has been confirmed by comparative investigations across a variety of plant taxa, proving *WUS* as an essential molecular regulator of plant development and reproductive functions.

### 3.3 WUSCHEL in Stress Response and Adaptation

WUS TFs are traditionally known for their role in stem cell maintenance and SE in plants, but recent studies indicate their involvement in plant stress response and adaptation. For example; *Arabidopsis* mutants exhibiting enhanced *WUS* expression, specifically *clv3‐2* and *clv1 bam1*, displayed improved salt tolerance as a result of sustained SAM proliferation [95]. This tolerance phenotype was attributable to the maintenance of actively dividing stem cells, rather than the transcriptional upregulation of stress markers, which is a characteristic of conventional stress‐responsive genes. Consequently, this observation underscores a developmental pathway, mediated by WUS, that facilitates salt adaptation. Building on this basis, a study reported that when plants are subjected to salt stress, METHIONINE SYNTHASE 2 (AtMS2) interacts with WUS/WOX proteins. *WUS* transcription is subsequently decreased by this interaction. *Atms2* mutants, on the other hand, had enhanced salt tolerance, demonstrating that variations in *WUS* expression have a direct impact on plants' ability to withstand salt stress [96]. Likewise, Liu et al., [97] found that RID2's role in 5′‐cap methylation (m^7^G modification) stabilizes WUS mRNA when exposed to heat stress. This prevents the transcript from breaking down, which allows *WUS* to continue functioning at high temperatures. These findings together show that WUS protects the meristem from environmental stress by acting during both the transcription and posttranscription stages. Consequently, *WUS* and *CLV3* exhibit dynamic alterations in their spatial expression and activity in reaction to fluctuations in ambient temperature, which correlate with modifications in the morphology and organization of SAM cells. A demonstration through 3D live imaging and computational modeling that this temperature‐dependent modulation of *WUS* promoter activity reflects the meristem's inherent capacity to govern growth and developmental homeostasis across diverse environmental conditions [98].

Although WUS‐focused reports remain limited, comparative studies of its WOX homologs indicate conserved stress‐responsive roles. For instance, *OsWOX13* enhances drought tolerance and accelerates flowering in rice [89]. Furthermore, *PagWOX11/12a* promotes root elongation and ROS scavenging in poplar [99], and *MdWOX13‐1* in apple increases drought resistance by improving ROS detoxification [100]. Consequently, these observations collectively demonstrate a conserved WUS/WOX regulatory module that integrates developmental regulation with environmental adaptation.

## 4 Biotechnological Application of WUS in Crop Improvement

### 4.1 WUS‐Centered Genome Engineering Strategies for Crop Improvement

**FIGURE 5:**
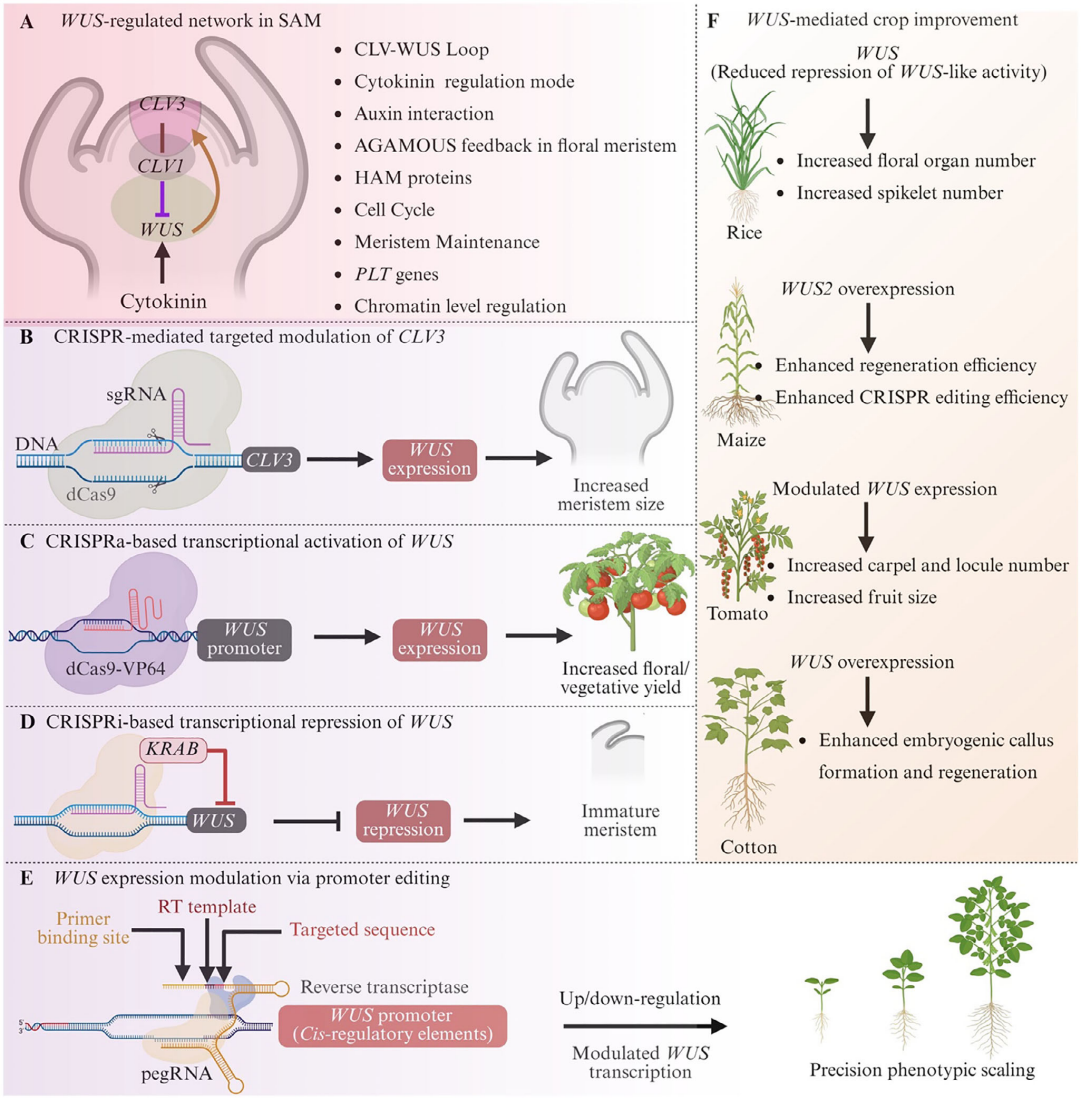
**CRISPR‐based modulation of the WUSCHEL (*WUS*) regulatory network for meristem engineering and crop improvement**. (**A)** Schematic representation of the WUS regulatory network in the shoot apical meristem (SAM). (**B)** CRISPR‐mediated attenuation of *CLV3* signaling alters the CLAVATA‐WUSCHEL (CLV–WUS) feedback loop, indirectly enhancing *WUS* activity and leading to increased meristem size. (**C)** Programmable transcriptional activation of WUS (CRISPRa) using dCas9‐VP64 targeted to the *WUS* promoter enhances *WUS* expression and promotes meristem activity. (**D)** Programmable transcriptional repression of *WUS* (CRISPRi) using dCas9‐KRAB reduces *WUS* transcription, resulting in decreased meristem size. (**E)** *WUS* transcription can be fine‐tuned through targeted modulation of WUS expression through promoter editing (e.g., prime editing of *cis*‐regulatory regions), which results in regulated meristem size modification. (**F)** Improved floral organ and spikelet number in rice, improved regeneration and genome editing efficiency in maize, increased locule number and fruit size in tomatoes, and improved embryogenic callus (EC) formation and regeneration in cotton are examples of crop improvement outcomes brought about by WUS pathway modulation.

*WUS* functions as a dosage‐sensitive organizer of stem cell maintenance in the SAM. Genetic research on *Arabidopsis* revealed that the absence of *WUS* activity leads to premature meristem termination caused by stem cell depletion [101], while ectopic or constitutive overexpression triggers aberrant stem cell proliferation and fasciated growth [102]. The CLAVATA–WUS negative feedback loop also limits *WUS* expression to the organizing center. When *CLV* signaling is disrupted, the *WUS* domain grows and the meristem gets bigger [103]. As shown in Figure 5A, *WUS* activity is part of a complex network of controls that includes CLV peptides, cytokinin signaling, chromatin regulation, and upstream repressors. These initial investigations demonstrate that *WUS* must be statistically and spatially constrained for proper development. So, for it to be useful in crop biotechnology, it needs to be able to change the dosage and regulatory domain of expression exactly, not just overexpress or completely knock it out.

#### 4.1.1 Modulating the CLV‐WUS Feedback Module

The CLV–WUS signaling circuit forms a conserved negative feedback loop that quantitatively regulates meristem size by limiting the spatial and transcriptional domain of *WUS*. WUS facilitates stem cell maintenance in the organizing center, but *CLV3*‐mediated signaling inhibits *WUS* expression in the central zone; thus, the regulated reduction of *CLV* signaling is a direct and mechanistically foreseeable approach to enhance meristem activity. Therefore, the practical engineering of meristem size should prioritize the modulation of feedback intensity above the disruption of route integrity.

Fundamental genetic investigations in *Arabidopsis* revealed that weak *clv3* alleles cause the enlargement of the SAM through the expansion of the *WUS* expression domain, leading to an increased number of floral organs without total meristem disorganization [103, 104]. These experiments demonstrated the causal relationship depicted in Figure 5B less CLV‐mediated repression allows for increased *WUS* activity, which correspondingly expands the stem cell population. Significantly, phenotypic severity is associated with the extent of repression loss, highlighting that dosage sensitivity in the feedback loop regulates developmental outcomes. This quantitative principle is maintained in crops. Mutations in *FLORAL ORGAN NUMBER* (*FON*) genes in rice, which are orthologs of CLV pathway components, lead to expanded FMs and an increased number of organs, illustrating the functional conservation of *CLV*‐mediated inhibition of *WUS*‐like activity in monocots [105]. These data suggest that a slight reduction in feedback repression can improve meristem production across evolutionary lineages. More recently, precision genome editing has enabled implementation of this principle in agronomic contexts. In maize, regulated repression of *WUS* was directly linked to yield‐related features using CRISPR‐Cas editing of CLE peptide genes within the CLV–WUS pathway, which changed the size of the inflorescence meristem and increased the number of kernel rows [106]. Instead of eliminating *CLV* signaling, these modifications decreased transcriptional output, thus partially alleviating inhibition on WUS pathway activity. The resultant phenotypes directly associate calibrated feedback attenuation with yield‐related variables.

These findings collectively illustrate that the CLV–WUS module functions as an adjustable developmental rheostat. Figure 5B illustrates that a reduction in *CLV* signaling strength broadens the *WUS* domain, increases the meristem size, and boosts organ formation. The principal operational tenet derived from this research is that effective WUS‐centered crop engineering necessitates quantitative regulation of feedback intensity instead of binary knockout approaches. This calibrated method reduces pleiotropic flaws while facilitating predictable modifications in meristem size and yield potential.

#### 4.1.2 Programmable Transcriptional Modulation for Temporal Control

Unlike permanent genome edits, programmable transcriptional regulation enables reversible and dosage‐sensitive control of endogenous gene expression. Catalytically inactive Cas9 (dCas9) platforms conjugated with transcriptional activators or repressors have been effectively utilized in plants to modulate native loci without modifying the DNA sequence. CRISPR‐Act3.0 and analogous activation systems exhibited significant transcriptional activation of endogenous genes in rice and *Arabidopsis*, whereas dCas9‐based repression modules accomplished targeted gene suppression across many plant species [107, 108]. These studies demonstrate the technical viability of programmable bidirectional transcriptional regulation in agricultural settings.

While direct modulation of *WUS* via CRISPRa/CRISPRi has not been documented, the biological characteristics of WUS substantiate its appropriateness for temporal transcriptional engineering. *WUS* acts as a dosage‐sensitive regulator of stem cell maintenance, and prolonged overexpression results in ectopic meristem formation and developmental anomalies [102]. These observations suggest that *WUS* activity requires stringent regulation in both intensity and duration, rendering it a suitable target for reversible regulatory strategies instead of persistent misexpression.

Figure 5C demonstrates that transient CRISPRa‐mediated activation of endogenous *WUS* can be utilized during early developmental or regeneration stages to enlarge the organizing center and promote stem cell proliferation. After this induction phase, CRISPRi‐mediated repression (Figure 5D) may diminish *WUS* transcription to reestablish meristem homeostasis and avert extended stem cell over proliferation. This bidirectional control framework facilitates temporal differentiation between regenerative enhancement and architectural stabilization.

Significantly, this programmable modulation is fundamentally distinct from the knockout or promoter editing strategies outlined in Sections 4.1.1 and 4.1.3. Transcriptional modulation enables dynamic and reversible adjustments of WUS output in response to developmental stages or biotechnological requirements, rather than permanently modifying feedback strength or *cis*‐regulatory architecture. Thus, incorporating *WUS*‐centered transcriptional regulation with existing plant CRISPRa/CRISPRi systems constitutes an effective and scalable approach for precisely modulating stem cell dynamics while reducing pleiotropic risk.

#### 4.1.3 *Cis*‐Regulatory Engineering of WUS and Network Components

Editing *cis*‐regulatory elements allows for quantitative tuning of *WUS* transcription instead of changing coding sequences. According to Rodríguez‐Leal et al. [109], CRISPR‐mediated promoter editing of *SlWUS* and *SlCLV3* in tomatoes produced a continuum of weak alleles that improved fruit morphology and expression levels, successfully re‐creating natural quantitative trait loci without serious pleiotropic defects. Enhancer editing of the duplicated *ZmWUS1‐B* locus in maize showed that differential RESPONSE REGULATOR binding motif counts resulted in additive effects on ear development and *WUS* expression [106]. These investigations offer concrete experimental proof that *WUS* dosage and associated meristem output are determined by *cis*‐element architecture. A workable method for achieving predictable changes in meristem size and controlled modulation of *WUS* transcription is promoter or enhancer editing, as illustrated in Figure 5E.

#### 4.1.4 Targeting Upstream Repressors of WUS Activity

The activity of *WUS* in the SAM is not only regulated by the strength of its own promoter, but also by a complex network of upstream repressors that limit stem cell maintenance both spatially and quantitatively. Strategic manipulation of these negative regulators offers an indirect yet potent method to refine WUS output without persistent overexpression. In the CLAVATA–WUSCHEL signaling pathway of *Arabidopsis*, the stem cell–derived peptide CLV3 activates receptor complexes (CLV1/CLV2) that inhibit *WUS* expression in the organizing centre [110]. Genetic attenuation of *CLV* signaling increases the *WUS* domain and enlarges meristems, suggesting that regulated reduction of upstream repression can augment meristem activity without direct WUS overexpression. Similarly, *ZmWUS1* expression in maize is restricted by orthologous CLAVATA pathway components including FEA2 and associated receptor‐like kinases [111]. Meristem expansion and a rise in the number of floral organs are caused by mutations in these regulators, further demonstrating how sensitive WUS production is to changes in upstream inhibitory signals. These results support targeting CLV‐like repressors as a logical engineering entry point and demonstrate conserved regulatory logic across species.

In addition to peptide‐receptor signaling, transcriptional corepressors contribute to restricting *WUS* expression domains. Members of the TOPLESS (TPL) family and associated corepressor complexes engage with meristem‐associated transcription factors to precisely regulate developmental gene networks [112]. Modifying the activity or recruitment of these corepressors can indirectly alleviate repression on WUS‐associated regulatory circuits, facilitating a controlled augmentation of stem cell maintenance.

Significantly, network‐level interventions operate mechanistically distinct from direct *WUS* overexpression (as elaborated in Section 4.2). Constitutive activation of *WUS* can induce regeneration, whereas upstream repression enables nuanced modulation of intrinsic regulatory circuits. This systems‐oriented approach diminishes developmental instability and enhances the preservation of meristem patterning integrity. Figure 5A demonstrates that targeting upstream repressors, including peptide‐mediated signaling components, receptor kinases, or transcriptional corepressors, broadens the engineering toolkit beyond the overexpression of individual genes. Utilizing conserved feedback circuits that inherently regulate *WUS* activity, crop enhancement strategies can attain improved meristem productivity with increased spatial and temporal accuracy.

#### 4.1.5 Network Level Editing and De Novo Domestication

*WUS*‐centered engineering has also been used in multiplex genome editing methods during quick domestication. Editing *SlWUS*, *SlCLV3*, *SELF PRUNING* (*SP*), and *COMPOUND INFLORESCENCE* (*S*) all at the same time in tomatoes made the plant's structure more compact, made the fruit bigger, and changed the number of organs in wild relatives [113, 114]. These interventions show that changing the WUS‐dependent meristem determinacy can be combined with other architectural regulators to get consistent agronomic results. Figure 5F shows that changing *WUS* activity leads to changes in the number of organs, yield components, and regeneration capacity across species such as in rice [105], maize [115, 116], tomato [109, 117] and cotton [64]

**FIGURE 6:**
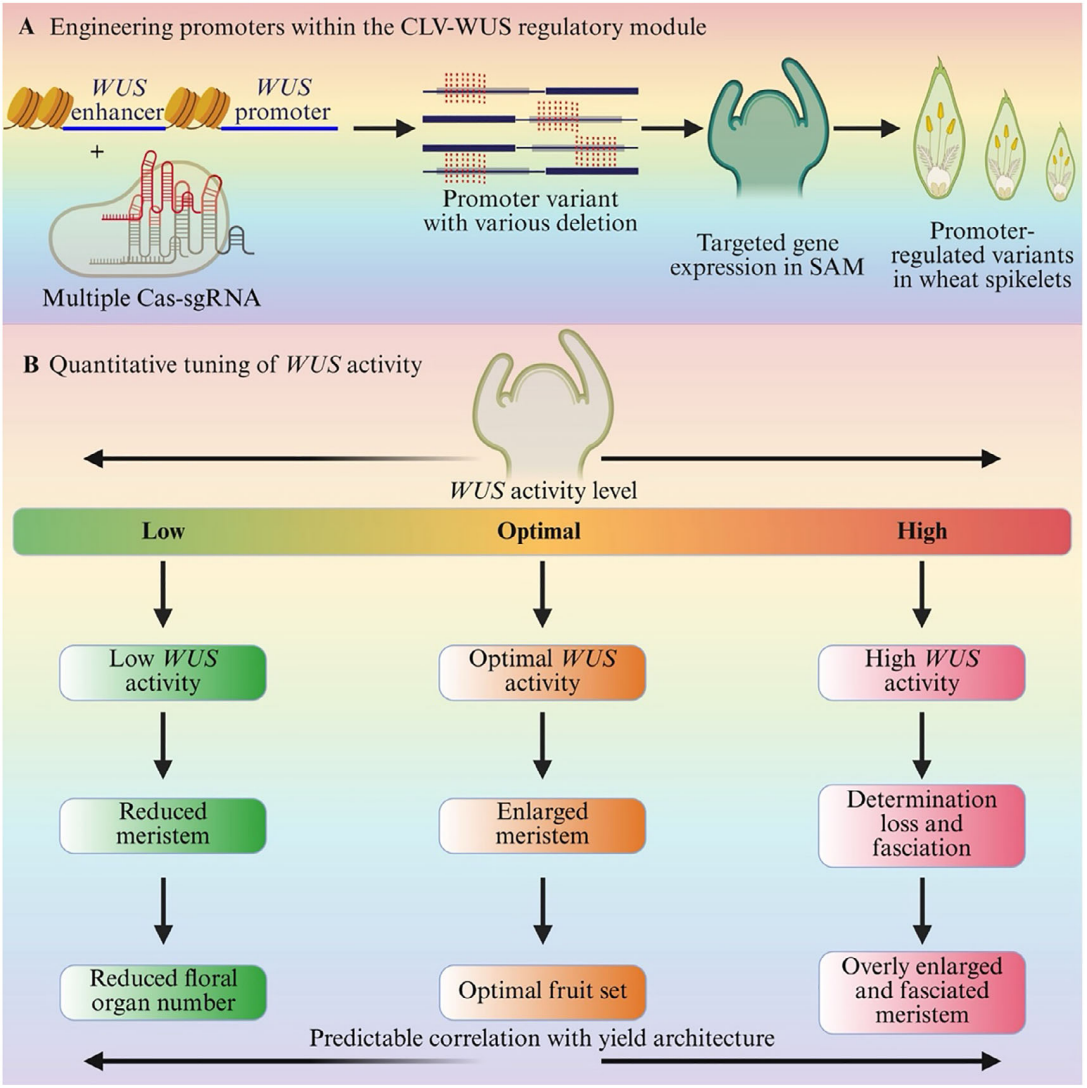
***Cis*‐regulatory engineering and quantitative tuning of WUSCHEL (WUS) activity define meristem function and yield architecture. (A)** CRISPR‐based *cis*‐regulatory engineering of the CLV–WUS module, where targeted editing of WUS promoter and enhancer elements generates quantitative promoter variants, enabling precise modulation of WUS expression in the shoot apical meristem (SAM) and subsequent variation in spikelet development. (**B)** WUS activity and developmental output have a quantitative, dosage‐dependent connection that shows that low WUS activity limits meristem growth, optimal levels support balanced organ formation and production, and high WUS activity causes meristem fasciation and developmental abnormalities.

Collectively, these studies establish a causal and implementation‐oriented framework: calibrated alteration of *WUS* dosage or its regulatory constraints leads to defined changes in meristem size, which in turn modifies organ production and yield‐related traits. By establishing *WUS* as a quantitatively engineerable developmental node instead of a generic editing target, genome engineering strategies can be systematically formulated to attain anticipated crop enhancement results. The quantitative and nonlinear relationship between *WUS* activity and agronomic output is summarized in Figure 6.

The aforementioned methodologies demonstrate how the modification of *WUS* activity or its regulatory network can be utilized to modify meristem size, organ quantity, and yield‐related traits in crops. In these contexts, *WUS* primarily functions as a developmental regulator, with its dosage and regulatory thresholds determining the architectural outcomes. *WUS* is crucial for maintaining meristem stability and has been demonstrated to be a potent morphogenic factor that can transform somatic cells into cells capable of generating embryos or meristems. This characteristic has increasingly been utilized in plant transformation systems to circumvent regeneration issues that complicate genome editing in numerous crop species. Consequently, *WUS* serves as both a focal point for developmental engineering and an effective instrument for enhancing transformation efficiency and facilitating genome editing in otherwise challenging germplasm.

### 4.2 WUS as a Regeneration‐Enhancing Tool in Transformation Systems

Efficient plant regeneration continues to be a significant obstacle in genome engineering, especially in genotype‐dependent or recalcitrant crops. In contrast to general morphogenic regulators, WUS operates as a dosage‐sensitive organizer of stem cell identity, rendering it particularly appropriates for the precise manipulation of regenerative capacity. Instead of functioning as a universal enhancer of transformation, *WUS* operates as an adjustable developmental switch: calibrated induction increases meristematic potential, while prolonged overexpression disrupts organ patterning. Consequently, the effective implementation of *WUS* necessitates regulated modulation of expression levels, spatial domains, and temporal durations.

Recent advancements in crop transformation elucidate that regeneration efficiency is contingent not solely on the presence of *WUS*, but on the precise engineering of its activity within developmental contexts. This idea is the basis for the following ways to put it into action.

**FIGURE 7:**
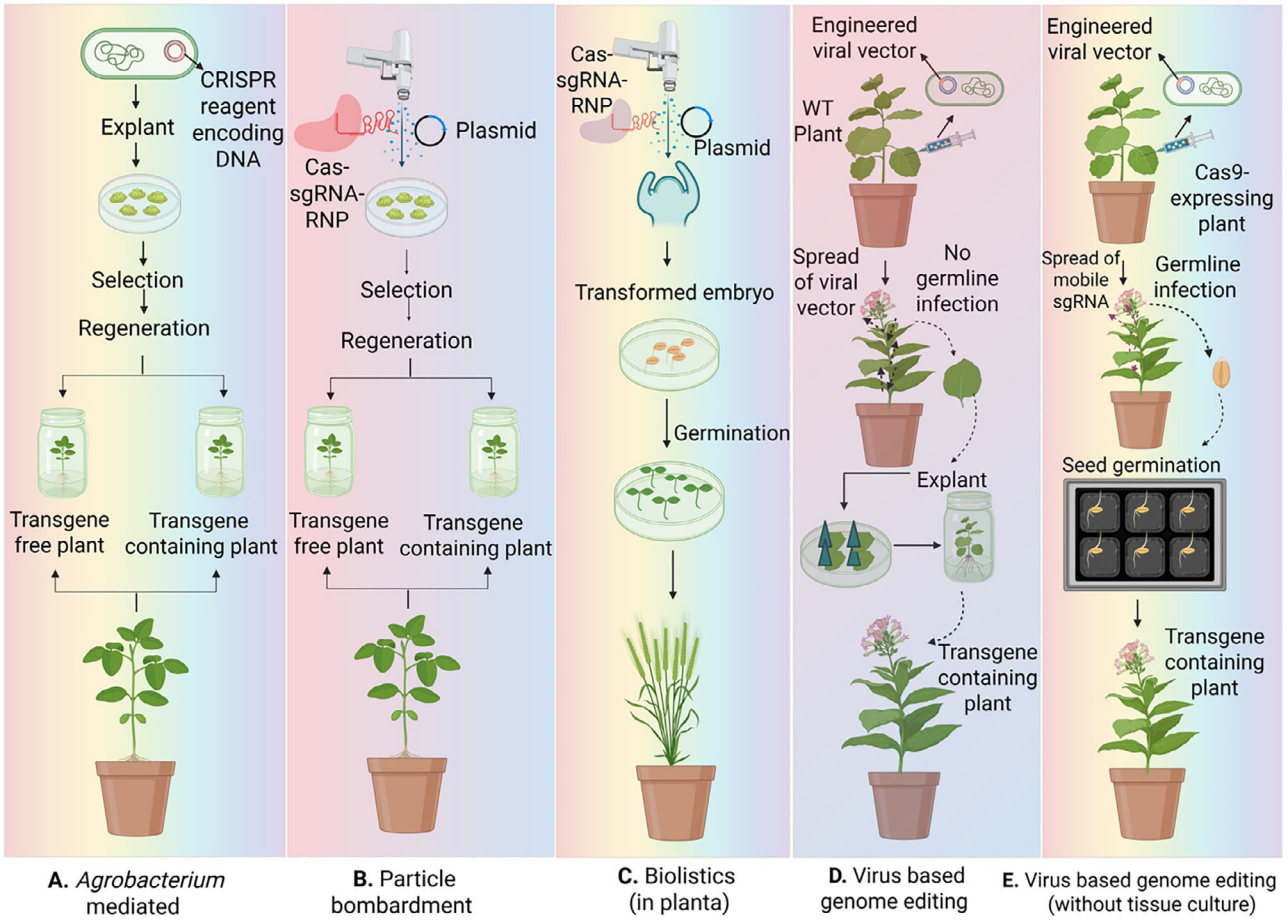
**Major genome‐editing delivery platforms used in plant transformation**. (**A)** *Agrobacterium*‐mediated transformation: Plasmids containing editing tools are introduced into calli; transgene‐free edited plants are made possible by transient expression in the absence of selection, while stable integration happens with selection. (**B)** Biolistic transformation: RNP or DNA‐coated microparticles are injected into cells; transgene integration may result from plasmid delivery with selection, whereas transgene‐free plants are typically produced by RNP delivery. (**C)** In planta biolistic transformation – The shoot apical meristem (SAM) of embryos is directly exposed and targeted with DNA/RNP‐coated particles, producing edited plants without tissue culture. (**D)** Virus‐induced genome editing (VIGE) with tissue culture – Viral vectors carrying Cas9 and gRNAs are infiltrated into leaves, causing edits in somatic cells; whole plants are regenerated via tissue culture. (**E)** Viral vectors transport tissue culture‐free VIGE‐sgRNAs fused to FT‐mobile RNA into Cas9 plants, where they enter germline cells and cause heritable changes in seeds.

Genome editing reagents can be introduced into plant cells via various established transformation methods, including Agrobacterium‐mediated transformation, particle bombardment, biolistic delivery of ribonucleoprotein complexes, and virus‐based genome editing systems. Each platform varies in its method of DNA or ribonucleoprotein delivery, integration characteristics, and regeneration needs. *Agrobacterium*‐mediated techniques are the predominant approach for stable transformation in numerous crops, while particle bombardment and RNP‐based biolistics facilitate transgene‐free editing through the delivery of preassembled Cas–sgRNA complexes. Recently, engineered viral vectors have facilitated the systemic dissemination of editing components and, in certain instances, genome editing without the need for tissue culture. The principal delivery frameworks are encapsulated in Figure 7 and delineate the technological context for the implementation of WUS‐mediated regeneration strategies.

#### 4.2.1 Dosage‐Dependent and Spatially Restricted WUS Induction

The maize *Wus2*–*Bbm* platform gives a clear structure for how to put things into action. Lowe et al. [115] showed that temporarily overexpressing *ZmWus2* and *ZmBbm* combined caused SE in maize genotypes that had been resistant to it before, such as PHH5G. Significantly, regeneration effectiveness was associated with the intensity of *Wus2* expression, suggesting a quantitative correlation between WUS dose and embryogenic competence.

Subsequent enhancements utilized tissue‐specific promoters, including *Zm‐PLTPpro* and *Zm‐Axig1pro* [116], thereby confining *Wus2* activity to the initial stages of embryogenesis. This spatial constraint‐maintained regeneration efficacy while averting pleiotropic developmental anomalies. These studies delineate a fundamental design principle: regeneration efficiency correlates positively with transient *WUS* activation, whereas stable constitutive expression is detrimental. Consequently, *WUS* manipulation in transformation systems is not binary (on/off), but rather quantitatively calibrated.

These findings indicate that the implementation of *WUS* should be customized to the specific crop context. In highly recalcitrant monocots, enhanced yet temporary *WUS* induction, in conjunction with *BBM*, is beneficial for surmounting genotype dependency. On the other hand, species that are likely to have developmental problems are better off with promoter‐restricted or low‐intensity induction strategies. So, for the best results in regeneration, *WUS* activity needs to be measured in numbers, not just overexpressed all the time. All of this data shows that dosage control and spatial restriction are the most important engineering principles for *WUS*‐based regeneration platforms.

#### 4.2.2 Transient and Nonintegrating WUS Systems

To avoid permanent developmental perturbation, dual‐delivery strategies were developed in maize [118, 119]. In these systems, *Wus2* is provided by a distinct *Agrobacterium* strain or expressed transiently without genomic incorporation. This enables *WUS* to act as a transient morphogenic inducer while guaranteeing its elimination in the final plant. The Non‐Integrating *Wus2* (NIW) system markedly enhanced transformation frequencies in recalcitrant lines such as B73 without stable retention of *Wus2* [119]. This delineates a secondary implementation guideline: *WUS* should operate as a temporary regenerative stimulus instead of a heritable transgene.

These strategies distinguish developmental reprogramming from the ultimate trait genotype, thereby addressing biosafety and regulatory issues. Crucially, they synchronize *WUS‐*based regeneration with advanced genome‐editing protocols, wherein temporary developmental activation is favored to preserve the genetic stability of modified lines.

#### 4.2.3 WUS‐Driven De Novo Meristem Formation for in Planta Editing

**FIGURE 8:**
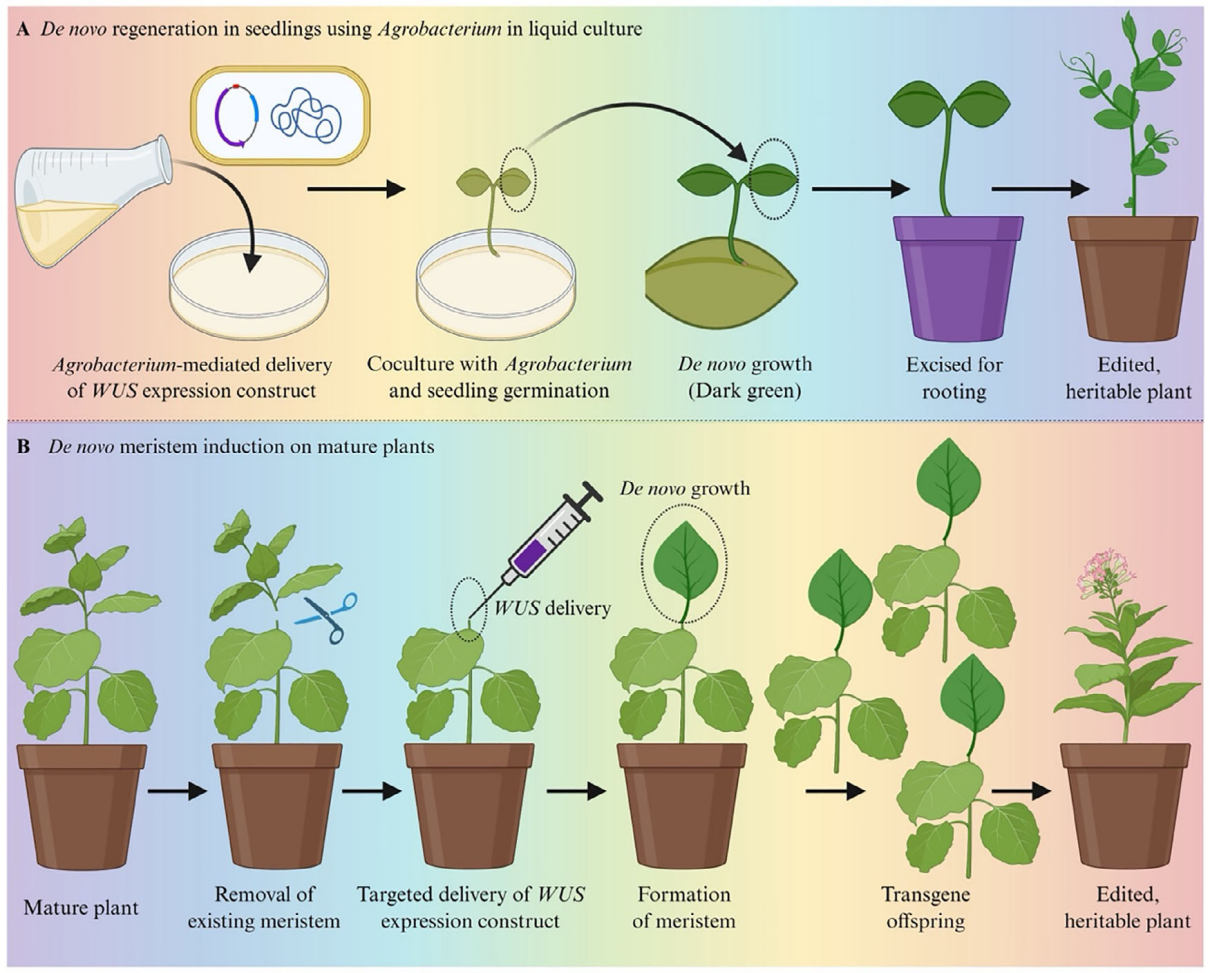
**WUS‐mediated regeneration strategies enabling genome editing. (A)** Induction of meristematic outgrowths in seedlings following *Agrobacterium*‐mediated delivery of a *WUS* expression construct. (**B)** Generation of edited, heritable shoots from soil‐grown plants via localized WUS induction in apical or axillary meristems.

WUS‐mediated meristem induction can occur either in the early stages of seedling development or directly in mature plants, contingent upon the delivery context and the developmental accessibility of the target tissues. Beyond tissue culture, combinatorial expression of *Wus2* with *STM* or *IPT* can induce de novo shoot meristems at wound sites [120, 121]. This approach bypasses callus formation and directly generates edited shoots in soil‐grown plants (Figure 8). Mechanistically, localized activation of *WUS* re‐establishes a domain akin to an organizing center, initiating stem cell identity programs in differentiated tissues. The result is foreseeable: proliferation of meristematic cell populations succeeded by shoot organogenesis. In this context, *WUS* serves not as a supportive element but as the causal agent for the establishment of the regenerative niche.

The incorporation of WUS into genome‐editing delivery systems enhances this concept. In systems utilizing temporary editing components, such as ribonucleoprotein‐based methods and combinatorial regeneration‐editing procedures, precise *WUS* activation improves shoot recovery without necessitating stable integration of morphogenic genes. Consequently, *WUS* operates both as a catalyst for development and as an enabler of effective genome engineering. Evidence from several crop systems further substantiates the significance of *WUS* and associated morphogenic regulators in enhancing transformation efficiency and regeneration competence across different species. A summary of representative instances of *WUS*‐mediated or *WUS*‐associated regeneration techniques in various crops is presented in Table 3.

**TABLE 3:** The role of different morphogenic factors in crop improvement.

Gene	Crop	Function	Editing tool/methods	Phenotypic marker	Remarks	References
*AtWUS*	*Coffea canephora*	Promotes dedifferentiation of somatic cells, leading to the formation of calli.	Transgenic overexpression	Formation of calli	Enhanced somatic embryogenesis (SE).	[74]
*WUS2*	*Sorgum bicolor*	Enhances transformation efficiency and regeneration capacity.	CRISPR‐Cas via *Agrobacterium*‐mediated transformation.	Enhanced regeneration of fertile plants across different sorghum genotypes	Supports genome editing by improving cell‐to‐plant transformation steps.	[122]
*EgWOX1–EgWOX9*	*Eucalyptus grandis and E. urophylla× E. grandis*	Regulate meristem development and regeneration.	External application of *WUS*‐derived peptides	Enhanced transformation efficiency	*WUS* peptides activated key metabolic pathways	[123]
*BrrWUSa*	*Brassica rapa* var. rapa	Enhances transformation and regeneration efficiency in turnip tissue culture.	CRISPR‐Cas targeted gene editing of BrrTCP4b	*BrrWUS*a: enhanced regeneration capacity. *BrrTCP4b*: Edited seedlings displayed an increase in leaf trichome number.	High regeneration efficiency without causing developmental defects in the transgenic plants.	[124]
*ZmBBM and ZmWUS*	*T. aestivum*	Acts as morphogenic regulators to enhance wheat transformation efficiency.	*Agrobacterium* mediated Transformation using BBM‐WUS construct	Fluorescent marker expression	Reduces transformation time and increases efficiency.	[125]

From a translational standpoint, calibrated *WUS*‐based systems broaden the spectrum of transformable elite germplasm, enhance recovery rates of edited events, and reduce regeneration timelines. By separating developmental reprogramming from the integration of stable traits, these strategies expedite breeding processes while preserving phenotypic stability in the final cultivar. Consequently, *WUS* manipulation functions both as a regeneration facilitator and as a foundational component for scalable genome engineering in agriculturally significant crops.

## 5 Conclusion and Future Prospective

WUS is a master regulator of stem cell fate in the SAM, with roles extending to SE, reproductive development, organ growth, and stress adaptation across diverse crops, including cereals, legumes, and woody bamboos. Its function as a morphogenic factor enables enhanced in vitro regeneration, overcoming key bottlenecks in plant transformation and facilitating CRISPR‐Cas‐mediated genome editing. Mechanistic insights into *WUS* regulation via *CLV* signaling, auxin crosstalk, and epigenetic modification, combined with multi‐omics, synthetic biology, and AI‐guided predictive modelling, provide a framework for translating fundamental knowledge into practical crop improvement. By anticipating off‐target effects, creating ideal guide RNAs, and aiding in the creation of novel Cas variants that target regulatory elements of WUS pathways, AI in particular can improve the precision of CRISPR‐Cas systems. Complex regulatory networks governing SE and meristem maintenance are further revealed by integrative analysis of multi‐omics datasets.

There are still issues such as the regulatory network of WUS is complicated and not fully understood in non‐model species, context‐dependent *WUS* expression can result in ectopic meristems or growth defects, and accurate spatiotemporal control is still challenging. Additionally, genotype‐dependence, delivery, and efficiency issues plague genome‐editing platforms. Future approaches should concentrate on species‐specific functional characterization, synthetic gene circuits that mimic WUS pathways, and spatiotemporally controlled *WUS* expression. Higher accuracy, repeatability, and efficiency are promised through integration with cutting‐edge genome‐editing technologies, such as base editing, prime editing, integrase‐based systems, and AI‐guided optimization. By connecting stem cell biology and applied agricultural innovation, this integrative approach places *WUS* as a key molecular lever for next‐generation crop improvement.

## Author Contributions

Conceptualization – ZA, MR, RKV, B.P. and QW. Funding acquisition – QW, ZA, B.P., and MR. Writing – original draft – ZA, MR, and LD, Software – ZA, MR, LD, and SR. Writing – review and editing – ZA, MR, LD, RKV, SR, AS, B.P. and QW.

## Funding sources

The preparation of this review was supported by grants from the National Natural Science Foundation of China (32471977 and 32071848); a grant from the Natural Science Foundation of Jiangsu Province (BK20231289); the Young Foreign Talent Program (Y20240114 and QN2022014012L). The support of the Metasequoia Faculty Research Start‐Up Funding (163100036, 163100028); the Natural Science Foundation For Distinguished Young Scholars of Nanjing Forestry University (JC2019004); the Project for Ground breaking Achievements of Nanjing Forestry University (202211) At the Bamboo Research Institute, Nanjing Forestry University, are also appreciated by the authors.

## Conflicts of Interest

Competing interests: The authors declare no competing interests. The selection of the handling editor and peer reviewers was carried out independently by the journal. The authors were not involved in the editorial or peer‐review process and were not aware of the identities of the reviewers.

## Supporting information




**Supporting Information**: advs75044‐sup‐0001‐SuppMat.pdf

## Data Availability

The author has nothing to report.

## References

[1] K. F. Mayer , H. Schoof , A. Haecker , M. Lenhard , G. Jürgens , and T. Laux , “Role of WUSCHEL in Regulating Stem Cell Fate in the Arabidopsis Shoot Meristem,” Cell 95 (1998): 805–815, 10.1016/S0092-8674(00)81703-1. 9865698

[2] F. L. Lopes , C. Galvan‐Ampudia , and B. Landrein , “WUSCHEL in the Shoot apical meristem: Old Player, New Tricks,” Journal of Experimental Botany 72 (2021): 1527–1535, 10.1093/jxb/eraa572. 33332559

[3] M. Somssich , B. I. Je , R. Simon , and D. Jackson , “CLAVATA‐WUSCHEL Signaling in the Shoot Meristem,” Development 143 (2016): 3238–3248. 27624829 10.1242/dev.133645

[4] S. Xu , Y. Wang , S. Yang , et al., “The WUSCHEL‐related Homeobox Transcription Factor CsWOX3 Negatively Regulates Fruit Spine Morphogenesis in Cucumber ( Cucumis sativus L.),” Horticulture Research 11 (2024): uhae163, 10.1093/hr/uhae163. 39108588 PMC11298622

[5] Y. Yang , M. Sun , C. Yuan , et al., “Interactions between WUSCHEL‐ and CYC2‐Like Transcription Factors in Regulating the Development of Reproductive Organs in Chrysanthemum Morifolium,” International Journal of Molecular Sciences 20 (2019): 1276, 10.3390/ijms20061276. 30875718 PMC6471657

[6] Z. Lu , G. Shao , J. Xiong , et al., “MONOCULM 3, an Ortholog of WUSCHEL in Rice, Is Required for Tiller Bud Formation,” Journal of Genetics and Genomics 42 (2015): 71–78, 10.1016/j.jgg.2014.12.005. 25697101

[7] T. Suzaki , T. Toriba , M. Fujimoto , N. Tsutsumi , H. Kitano , and H.‐Y. Hirano , “Conservation and Diversification of Meristem Maintenance Mechanism in Oryza sativa: Function of the FLORAL ORGAN NUMBER2 Gene,” Plant and Cell Physiology 47 (2006): 1591–1602, 10.1093/pcp/pcl025. 17056620

[8] D. Rodríguez‐Leal , Z. H. Lemmon , J. Man , M. E. Bartlett , and Z. B. Lippman , “Engineering Quantitative Trait Variation for Crop Improvement by Genome Editing,” Cell 171 (2017): 470. 28919077 10.1016/j.cell.2017.08.030

[9] C. Xu , K. L. Liberatore , C. A. MacAlister , et al., “A Cascade of Arabinosyltransferases Controls Shoot Meristem Size in Tomato,” Nature genetics 47 (2015): 784–792, 10.1038/ng.3309. 26005869

[10] T. Greb and J. U. Lohmann , “Plant Stem Cells,” Current biology 26 (2016): R816. 27623267 10.1016/j.cub.2016.07.070

[11] Y. Ç. Ince and K. Sugimoto , “Illuminating the Path to Shoot Meristem Regeneration: Molecular Insights into Reprogramming Cells into Stem Cells,” Current Opinion in Plant Biology 76 (2023): 102452, 10.1016/j.pbi.2023.102452. 37709567

[12] E. Van Der Graaff , T. Laux , and S. A. Rensing , “The WUS Homeobox‐containing (WOX) Protein family,” Genome Biology 10 (2009): 248, 10.1186/gb-2009-10-12-248. 20067590 PMC2812940

[13] F. Du , Z. Chang , X. Kong , et al., “Functional Conservation and Divergence of the WOX Gene Family in Regulating Meristem Activity: from Arabidopsis to Crops,” Plant Physiology 199 (2025): kiaf374, 10.1093/plphys/kiaf374. 40857588

[14] P. Lindsay , K. W. Swentowsky , and D. Jackson , “Cultivating Potential: Harnessing Plant Stem Cells for Agricultural Crop Improvement,” Molecular Plant 17 (2024): 50–74. 38130059 10.1016/j.molp.2023.12.014

[15] G. Daum , A. Medzihradszky , T. Suzaki , and J. U. Lohmann , “A Mechanistic Framework for Noncell Autonomous Stem Cell Induction in Arabidopsis,” Proceedings of the National Academy of Sciences 111 (2014): 14619–14624, 10.1073/pnas.1406446111. PMC4210042 25246576

[16] A. C. Willoughby and Z. L. Nimchuk , “WOX Going on: CLE Peptides in Plant Development,” Current Opinion in Plant Biology 63 (2021): 102056, 10.1016/j.pbi.2021.102056. 34077886 PMC8545713

[17] F. Xu and D. Jackson , “Learning from CIK Plants,” Nature Plants 4 (2018): 195–196, 10.1038/s41477-018-0125-x. 29581512

[18] T. Q. Dao , N. Weksler , H. M.‐H. Liu , S. Leiboff , and J. C. Fletcher , Development (Cambridge, England) 149 (2022): dev200787. 36111520 10.1242/dev.200787

[19] Y. Zhou , A. Yan , H. Han , et al., “Hairy Meristem with WUSCHEL Confines CLAVATA3 Expression to the Outer Apical Meristem Layers,” Science 361 (2018): 502–506, 10.1126/science.aar8638. 30072538 PMC6095697

[20] H. Han , A. Yan , L. Li , et al., “A Signal Cascade Originated from Epidermis Defines Apical‐Basal Patterning of Arabidopsis Shoot Apical Meristems,” Nature Communications 11 (2020): 1214, 10.1038/s41467-020-14989-4. PMC7058014 32139673

[21] H. F. Hofhuis and R. Heidstra , “Transcription Factor Dosage: More or Less Sufficient for Growth,” Current Opinion in Plant Biology 45 (2018): 50–58, 10.1016/j.pbi.2018.05.008. 29852330

[22] M. Perales , K. Rodriguez , S. Snipes , R. K. Yadav , M. Diaz‐Mendoza , and G. V. Reddy , “Threshold‐Dependent Transcriptional Discrimination Underlies Stem Cell Homeostasis,” Proceedings National Academy of Science USA 113 (2016): E6298–E6306. 10.1073/pnas.1607669113 PMC5068294 27671653

[23] J. Schlegel , G. Denay , R. Wink , et al., “Control of Arabidopsis Shoot Stem Cell Homeostasis by Two Antagonistic CLE Peptide Signaling Pathways,” Elife 10 (2021): 70934, 10.7554/eLife.70934. PMC8594942 34643181

[24] J. Cammarata , C. Morales Farfan , M. J. Scanlon , and A. H. K. Roeder , “Cytokinin–CLAVATA Cross‐talk Is an Ancient Mechanism Regulating Shoot Meristem Homeostasis in Land Plants,” Proceedings of the National Academy of Sciences 119 (2022): 2116860119, 10.1073/pnas.2116860119. PMC9168927 35344421

[25] M. Xie , H. Chen , L. Huang , R. C. O'Neil , M. N. Shokhirev , and J. R. Ecker , “A B‐ARR‐Mediated Cytokinin Transcriptional Network Directs Hormone Coss‐Regulation and Shoot Development,” Nature Communications 9 (2018): 1604, 10.1038/s41467-018-03921-6. PMC5913131 29686312

[26] F. Zhang , A. May , and V. F. Irish , “Type‐B Arabidopsis Response Regulators Directly Activate WUSCHEL,” Trends in Plant Science 22 (2017): 815–817, 10.1016/j.tplants.2017.08.007. 28886911

[27] A. Leibfried , J. P. To , W. Busch , et al., “WUSCHEL Controls Meristem Function by Direct Regulation of Cytokinin‐Inducible Response Regulators,” Nature 438 (2005): 1172–1175, 10.1038/nature04270. 16372013

[28] Y. Ma , A. Miotk , Z. Šutiković , et al., “WUSCHEL Acts as an Auxin Response Rheostat to Maintain Apical Stem Cells in Arabidopsis,” Nature Communications 10 (2019): 5093, 10.1038/s41467-019-13074-9. PMC6841675 31704928

[29] W. J. Meng , Z. J. Cheng , Y. L. Sang , et al., “Type‐B ARABIDOPSIS RESPONSE REGULATORs Specify the Shoot Stem Cell Niche by Dual Regulation of WUSCHEL,” The Plant Cell 29 (2017): 1357–1372, 10.1105/tpc.16.00640. 28576846 PMC5502443

[30] F. Besnard , Y. Refahi , V. Morin , et al., “Cytokinin Signalling Inhibitory Fields Provide Robustness to Phyllotaxis,” Nature 505 (2014): 417–421, 10.1038/nature12791. 24336201

[31] G. Cui , Y. Li , L. Zheng , C. Smith , M. W. Bevan , and Y. Li , “The Peptidase DA1 Cleaves and Destabilizes WUSCHEL to Control Shoot Apical Meristem Size,” Nature Communications 15 (2024): 4627, 10.1038/s41467-024-48361-7. PMC11143343 38821962

[32] L. Geng , M. Tan , Q. Deng , et al., “Transcription Factors WOX11 and LBD16 Function with Histone Demethylase JMJ706 to Control Crown Root Development in Rice,” The Plant Cell 36 (2024): 1777–1790, 10.1093/plcell/koad318. 38190205 PMC11062443

[33] H. Wang , X. Tong , L. Tang , et al., “RLB (RICE LATERAL BRANCH) Recruits PRC2‐Mediated H3K27 Tri‐Methylation on OsCKX4 to Regulate Lateral Branching,” Plant Physiology 188 (2022): 460–476, 10.1093/plphys/kiab494. 34730827 PMC8774727

[34] V. Nguyen and R. Gutzat , “Epigenetic Regulation in the Shoot Apical Meristem,” Current Opinion in Plant Biology 69 (2022): 102267, 10.1016/j.pbi.2022.102267. 35985107

[35] W.‐H. Shen and L. Xu , “Chromatin Remodeling in Stem Cell Maintenance in Arabidopsis Thaliana,” Molecular plant 2 (2009): 600–609, 10.1093/mp/ssp022. 19825642

[36] W. Li , H. Liu , Z. J. Cheng , et al., “DNA Methylation and Histone Modifications Regulate De Novo Shoot Regeneration in Arabidopsis by Modulating WUSCHEL Expression and Auxin Signaling,” PLoS Genetics 7 (2011): 1002243, 10.1371/journal.pgen.1002243. PMC3158056 21876682

[37] J. Sloan , J. P. Hakenjos , M. Gebert , et al., “Structural Basis for the Complex DNA Binding Behavior of the Plant Stem Cell Regulator WUSCHEL,” Nature Communications 11 (2020): 2223, 10.1038/s41467-020-16024-y. PMC7203112 32376862

[38] L. Luo , L. Liu , L. She , et al., “DRN Facilitates WUS Transcriptional Regulatory Activity by Chromatin Remodeling to Regulate Shoot Stem Cell Homeostasis in Arabidopsis,” PLoS Biology 22 (2024): 3002878, 10.1371/journal.pbio.3002878. PMC11548754 39514478

[39] G. Wang , Z. Wu , and B. Sun , “KNUCKLES Regulates Floral Meristem Termination by Controlling Auxin Distribution and Cytokinin Activity,” The Plant Cell 37 (2024): koae312, 10.1093/plcell/koae312. 39576002 PMC11663560

[40] J. Zeng , X. Zhao , Z. Liang , et al., “Nitric Oxide Controls Shoot Meristem Activity via Regulation of DNA Methylation,” Nature Communications 14 (2023): 8001, 10.1038/s41467-023-43705-1. PMC10696095 38049411

[41] I. Bäurle and T. Laux , “Regulation of WUSCHEL Transcription in the Stem Cell Niche of the Arabidopsis Shoot Meristem,” The Plant Cell 17 (2005): 2271–2280, 10.1105/tpc.105.032623. 15980263 PMC1182488

[42] D. Haig , “Transposable Elements: Self‐Seekers of the Germline, Team‐Players of the Soma,” BioEssays 38 (2016): 1158–1166, 10.1002/bies.201600125. 27604404

[43] I. Pagán , N. Montes , M. G. Milgroom , and F. García‐Arenal , “Vertical Transmission Selects for Reduced Virulence in a Plant Virus and for Increased Resistance in the Host,” PLoS Pathogens 10 (2014): 1004293, 10.1371/journal.ppat.1004293. PMC4117603 25077948

[44] C. O. Ossai , M. O. Balogun , and N. G. Maroya , “Status and Prospects of Yam Somatic Embryogenesis: a Pathway for Biotechnology Applications,” In Vitro Cellular and Developmental Biology ‐ Plant 61 (2024): 560–570. 10.1007/s11627-023-10397-7 PMC12343703 40814591

[45] M. Ramakrishnan , M. Zhou , S. A. Ceasar , et al., “Epigenetic Modifications and miRNAs Determine the Transition of Somatic Cells into Somatic Embryos,” Plant Cell Reports 42 (2023): 1845–1873, 10.1007/s00299-023-03071-0. 37792027

[46] A. Kaur , A. Sharma , and S. K. Upadhyay , Plant Receptor‐Like Kinases (Elsevier, 2023), 149–166.

[47] M. Zhang , X. Chen , X. Lou , et al., “Identification of WUSCHEL‐related Homeobox (WOX) Gene family Members and Determination of Their Expression Profiles during Somatic Embryogenesis in Phoebe Bournei,” Forestry Research 3 (2023): 5. 39526263 10.48130/FR-2023-0005 PMC11524275

[48] J. Zuo , Q. Niu , G. Frugis , and N. Chua , “The WUSCHEL Gene Promotes Vegetative‐to‐Embryonic Transition in Arabidopsis,” The Plant Journal 30 (2002): 349–359, 10.1046/j.1365-313X.2002.01289.x. 12000682

[49] K. Boutilier , R. Offringa , V. K. Sharma , et al., “Ectopic Expression of BABY BOOM Triggers a Conversion from Vegetative to Embryonic Growth,” The Plant Cell 14 (2002): 1737–1749, 10.1105/tpc.001941. 12172019 PMC151462

[50] J. J. Harada , “Role of Arabidopsis LEAFY COTYLEDON Genes in Seed Development,” Journal of Plant Physiology 158 (2001): 405–409, 10.1078/0176-1617-00351.

[51] S. Yang , Q. Chen , and S. Liu , “FUSCA3, a Multi‐role Regulator in the Process of Plant Growth and Development,” Plant Cell, Tissue and Organ Culture (PCTOC) 150 (2022): 1–13, 10.1007/s11240-022-02243-2.

[52] Q. Zheng , Y. Zheng , and S. E. Perry , “AGAMOUS‐Like15 Promotes Somatic Embryogenesis in Arabidopsis and Soybean in Part by the Control of Ethylene Biosynthesis and Response,” Plant physiology 161 (2013): 2113–2127, 10.1104/pp.113.216275. 23457229 PMC3613480

[53] E. Valencia‐Lozano , J. L. Cabrera‐Ponce , A. Barraza , et al., “Editing of SlWRKY29 by CRISPR‐activation Promotes Somatic Embryogenesis in Solanum Lycopersicum Cv. Micro‐Tom,” PLoS ONE 19 (2024): 0301169, 10.1371/journal.pone.0301169. PMC10984418 38557903

[54] Y. Yang , N. Wang , and S. Zhao , “Functional Characterization of a WRKY family Gene Involved in Somatic Embryogenesis in Panax ginseng,” Protoplasma 257 (2020): 449–458, 10.1007/s00709-019-01455-2. 31760482

[55] Y. A. Purwestri , Y.‐S. Lee , C. Meehan , et al., “RWP‐RK Domain 3 (OsRKD3) Induces Somatic Embryogenesis in Black Rice,” BMC Plant Biology 23 (2023): 202, 10.1186/s12870-023-04220-z. 37076789 PMC10114336

[56] L. Xu , Y. Liu , J. Zhang , et al., “Genomic Survey and Expression Analysis of LcARFs Reveal Multiple Functions to Somatic Embryogenesis in Liriodendron,” BMC Plant Biology 24 (2024): 94, 10.1186/s12870-024-04765-7. 38326748 PMC10848544

[57] Y. Qin , B. Zhang , X. Luo , et al., “Development of an Agrobacterium Tumefaciens‐mediated Transformation System for Somatic Embryos and Transcriptome Analysis of LcMYB1's Inhibitory Effect on Somatic Embryogenesis in Litchi chinensis,” Journal of Integrative Agriculture 24 (2024): 594–609.

[58] J.‐S. Park , K. H. Park , S.‐J. Park , et al., “WUSCHEL Controls Genotype‐dependent Shoot Regeneration Capacity in Potato,” Plant Physiology 193 (2023): 661–676, 10.1093/plphys/kiad345. 37348867

[59] W. Zhang , X. Xie , L. Le , and F. Cao , “Transcriptional Profiling Reveals Key Regulatory Roles of the WUSCHEL‐Related Homeobox Gene Family in Yellowhorn (Xanthoceras sorbifolia Bunge),” Forests 15 (2024): 376.

[60] T. Zhu , P. N. Moschou , J. M. Alvarez , J. J. Sohlberg , and S. Von Arnold , “WUSCHEL‐RELATED HOMEOBOX 8/9 Is Important for Proper Embryo Patterning in the Gymnosperm Norway Spruce,” Journal of experimental botany 65 (2014): 6543–6552, 10.1093/jxb/eru371. 25205582 PMC4246185

[61] H. Ren , S. Chen , J. Hou , and H. Li , “Genome‐wide Identification, Expression Analyses of Wuschel‐related Homeobox (WOX) Genes in Brachypodium Distachyon and Functional Characterization of BdWOX12,” Gene 836 (2022): 146691, 10.1016/j.gene.2022.146691. 35738446

[62] A. Xu , J. Yang , S. Wang , et al., “Characterization and Expression Profiles of WUSCHEL‐related Homeobox (WOX) Gene family in Cultivated Alfalfa (Medicago sativa L.),” BMC Plant Biology 23 (2023): 471, 10.1186/s12870-023-04476-5. 37803258 PMC10557229

[63] W. Zheng , X. Zhang , Z. Yang , et al., “AtWuschel Promotes Formation of the Embryogenic Callus in Gossypium Hirsutum,” PLoS ONE 9 (2014): 87502, 10.1371/journal.pone.0087502. PMC3909107 24498119

[64] O. Bouchabké‐Coussa , M. Obellianne , D. Linderme , et al., “Wuschel Overexpression Promotes Somatic Embryogenesis and Induces Organogenesis in Cotton (Gossypium hirsutum L.) Tissues Cultured in Vitro,” Plant Cell Reports 32 (2013): 675–686, 10.1007/s00299-013-1402-9. 23543366

[65] A. Calabuig‐Serna , R. Mir , and J. M. Seguí‐Simarro , “Calcium Dynamics, WUSCHEL Expression and Callose Deposition during Somatic Embryogenesis in Arabidopsis Thaliana Immature Zygotic Embryos,” Plants 12 (2023): 1021, 10.3390/plants12051021. 36903882 PMC10005541

[66] J. Wang , T. Zhang , L. Ren , et al., “Establishment of a Direct Somatic Embryogenesis Regeneration System Using Immature Cotyledon Explants in Camellia Sinensis Cv. Shuchazao,” Industrial Crops and Products 210 (2024): 118076, 10.1016/j.indcrop.2024.118076.

[67] L. Lu , A. Holt , X. Chen , et al., “miR394 enhances WUSCHEL ‐Induced Somatic Embryogenesis in Arabidopsis thaliana,” New Phytologist 238 (2023): 1059–1072, 10.1111/nph.18801. 36751948

[68] X. Wei , M. Geng , J. Li , H. Duan , F. Li , and X. Ge , “GhWOX11 and GhWOX12 Promote Cell Fate Specification during Embryogenesis,” Industrial Crops and Products 184 (2022): 115031, 10.1016/j.indcrop.2022.115031.

[69] F. L. McFarland , R. Collier , N. Walter , B. Martinell , S. M. Kaeppler , and H. F. Kaeppler , “A Key to Totipotency: Wuschel‐like homeobox 2a Unlocks Embryogenic Culture Response in Maize ( Zea mays L.),” Plant Biotechnology Journal 21 (2023): 1860–1872, 10.1111/pbi.14098. 37357571 PMC10440991

[70] M. Haghighat , R. Zhong , and Z.‐H. Ye , “WUSCHEL‐RELATED HOMEOBOX Genes Are Crucial for Normal Vascular Organization and Wood Formation in Poplar,” Plant Science 346 (2024): 112138, 10.1016/j.plantsci.2024.112138. 38825043

[71] K. Zhang , R. Wang , H. Zi , et al., “AUXIN RESPONSE FACTOR3 Regulates Floral Meristem Determinacy by Repressing Cytokinin Biosynthesis and Signaling,” The Plant Cell 30 (2018): 324–346, 10.1105/tpc.17.00705. 29371438 PMC5868698

[72] W. Zhao , Z. Chen , X. Liu , et al., “Cs LFY Is Required for Shoot Meristem Maintenance via Interaction with WUSCHEL in Cucumber ( Cucumis sativus ),” New Phytologist 218 (2018): 344–356, 10.1111/nph.14954. 29274285

[73] A. Haecker , R. Groß‐Hardt , B. Geiges , et al., “Expression Dynamics of WOX Genes Mark Cell Fate Decisions during Early Embryonic Patterning in Arabidopsis thaliana,” 131 (2004): 657–668. 10.1242/dev.00963 14711878

[74] A. Arroyo‐Herrera , A. Ku Gonzalez , R. Canche Moo , et al., “Expression of WUSCHEL in Coffea Canephora Causes Ectopic Morphogenesis and Increases Somatic Embryogenesis,” Plant Cell, Tissue and Organ Culture 94 (2008): 171–180, 10.1007/s11240-008-9401-1.

[75] Y. H. Su , X. Y. Zhao , Y. B. Liu , C. L. Zhang , S. D. O'Neill , and X. S. Zhang , “Auxin‐Induced WUS Expression Is Essential for Embryonic Stem Cell Renewal during Somatic Embryogenesis in Arabidopsis,” The Plant Journal 59 (2009): 448–460, 10.1111/j.1365-313X.2009.03880.x. 19453451 PMC2788036

[76] J. Palovaara , H. Hallberg , C. Stasolla , and I. Hakman , “Comparative Expression Pattern Analysis of WUSCHEL‐Related Homeobox 2 ( WOX2 ) and WOX8 / 9 in Developing Seeds and Somatic Embryos of the Gymnosperm Picea Abies,” New Phytologist 188 (2010): 122–135, 10.1111/j.1469-8137.2010.03336.x. 20561212

[77] G. Gambino , M. Minuto , P. Boccacci , I. Perrone , R. Vallania , and I. Gribaudo , “Characterization of Expression Dynamics of WOX Homeodomain Transcription Factors during Somatic Embryogenesis in Vitis vinifera,” Journal of Experimental Botany 62 (2011): 1089–1101, 10.1093/jxb/erq349. 21127025

[78] C. Santa‐Catarina , R. R. De Oliveira , L. Cutri , E. I. S. Floh , and M. C. Dornelas , “WUSCHEL‐related Genes Are Expressed during Somatic Embryogenesis of the Basal Angiosperm Ocotea Catharinensis Mez. (Lauraceae),” Trees 26 (2012): 493–501, 10.1007/s00468-011-0610-6.

[79] S. B. Hassani , J.‐F. Trontin , J. Raschke , K. Zoglauer , and A. Rupps , “Constitutive Overexpression of a Conifer WOX2 Homolog Affects Somatic Embryo Development in Pinus Pinaster and Promotes Somatic Embryogenesis and Organogenesis in Arabidopsis Seedlings,” Frontiers in Plant Science 13 (2022): 838421, 10.3389/fpls.2022.838421. 35360299 PMC8960953

[80] L. Zhang , D. DeGennaro , G. Lin , J. Chai , and E. D. Shpak , “ERECTA family Signaling Constrains CLAVATA3 and WUSCHEL to the Center of the Shoot apical meristem,” Development (Cambridge, England) 148 (2021): dev189753, 10.1242/dev.189753. 33593817

[81] Y. Yu , M. Yang , X. Liu , et al., “Genome‐wide Analysis of the WOX Gene family and the Role of EjWUSa in Regulating Flowering in Loquat (Eriobotrya japonica),” Frontiers in Plant Science 13 (2022): 1024515, 10.3389/fpls.2022.1024515. 36407616 PMC9669421

[82] L. Dong , Y. Shi , P. Li , S. Zhong , Y. Sun , and F. Yang , “Constructing the Maize Inflorescence Regulatory Network by Using Efficient tsCUT&Tag Assay,” The Crop Journal 11 (2023): 951–956, 10.1016/j.cj.2022.11.004.

[83] Y. Sun , L. Dong , L. Kang , W. Zhong , D. Jackson , and F. Yang , “Progressive Meristem and Single‐cell Transcriptomes Reveal the Regulatory Mechanisms Underlying Maize Inflorescence Development and Sex Differentiation,” Molecular Plant 17 (2024): 1019–1037, 10.1016/j.molp.2024.06.007. 38877701

[84] R. Groß‐Hardt , M. Lenhard , and T. Laux , “WUSCHEL Signaling Functions in Interregional Communication during Arabidopsis Ovule Development,” Genes & Development 16 (2002): 1129–1138, 10.1101/gad.225202. 12000795 PMC186242

[85] V. K. Sharma and J. C. Fletcher , “Maintenance of Shoot and Floral Meristem Cell Proliferation and Fate,” Plant Physiology 129 (2002): 31–39, 10.1104/pp.010987. 12011335 PMC1540224

[86] C. E. Wong , S. Y. Khor , P. L. Bhalla , and M. B. Singh , “Novel Spatial Expression of Soybean WUSCHEL in the Incipient Floral Primordia,” Planta 233 (2011): 553–560, 10.1007/s00425-010-1320-9. 21116646

[87] W. Xiang , X. WANG , J. REN , M. A. Ying , and Y. I. N. Jun , “Identification of Differentially Expressed Genes in the Salivary Gand of Rhipicephalus Haemaphysaloides by the Suppression Subtractive Hybridization Approach,” Journal of Integrative Agriculture 11 (2012): 1528, 10.1016/S2095-3119(12)60153-1.

[88] Y. Ohmori , W. Tanaka , M. Kojima , H. Sakakibara , and H.‐Y. Hirano , “WUSCHEL‐RELATED HOMEOBOX4 Is Involved in Meristem Maintenance and Is Negatively Regulated by the CLE Gene FCP1 in Rice,” The Plant Cell 25 (2013): 229–241, 10.1105/tpc.112.103432. 23371950 PMC3584538

[89] P.‐T. Minh‐Thu , J. S. Kim , S. Chae , et al., “A WUSCHEL Homeobox Transcription Factor, OsWOX13, Enhances Drought Tolerance and Triggers Early Flowering in Rice,” Molecules and Cells 41 (2018): 781–798. 30078233 10.14348/molcells.2018.0203 PMC6125423

[90] T. R. Ramkumar , M. Kanchan , S. K. Upadhyay , and J. K. Sembi , “Identification and Characterization of WUSCHEL‐related Homeobox (WOX) Gene family in Economically Important Orchid Species Phalaenopsis Equestris and Dendrobium Catenatum,” Plant Gene 14 (2018): 37–45, 10.1016/j.plgene.2018.04.004.

[91] Y. Yasui , Y. Ohmori , Y. Takebayashi , H. Sakakibara , and H.‐Y. Hirano , “WUSCHEL‐RELATED HOMEOBOX4 Acts as a Key Regulator in Early Leaf Development in Rice,” PLoS Genetics 14 (2018): 1007365, 10.1371/journal.pgen.1007365. PMC5933814 29684018

[92] E. Honda , C.‐L. Yew , T. Yoshikawa , Y. Sato , K. Hibara , and J.‐I. Itoh , “LEAF LATERAL SYMMETRY1, a Member of the WUSCHEL‐RELATED HOMEOBOX3 Gene Family, Regulates Lateral Organ Development Differentially from Other Paralogs, NARROW LEAF2 and NARROW LEAF3 in Rice,” Plant and Cell Physiology 59 (2018): 376. 29272531 10.1093/pcp/pcx196

[93] Q. Hao , L. Zhang , Y. Yang , Z. Shan , and X. Zhou , “Genome‐Wide Analysis of the WOX Gene Family and Function Exploration of GmWOX18 in Soybean,” Plants 8 (2019): 215, 10.3390/plants8070215. 31373320 PMC6681341

[94] L. Tang , Y. He , B. Liu , Y. Xu , and G. Zhao , “Genome‐Wide Identification and Characterization Analysis of WUSCHEL‐Related Homeobox Family in Melon (Cucumis melo L.),” International Journal of Molecular Sciences 24 (2023): 12326, 10.3390/ijms241512326. 37569702 PMC10419029

[95] Y. S. Jun , O.‐K. Cha , J. H. Kim , and H. Lee , “Shoot Meristem Activity Is Involved in Salt Tolerance on Arabidopsis Shoot Growth,” Journal of Plant Biology 62 (2019): 410–418, 10.1007/s12374-019-0348-z.

[96] J. Qiu , M. Chen , F. Lu , X. Chen , Z. Cai , and T. Huang , “Methionine Synthase 2 Represses Stem Cell Maintenance of Arabidopsis Thaliana in Response to Salt Stress,” Plants 13 (2024): 2224, 10.3390/plants13162224. 39204660 PMC11359516

[97] S. Liu , H. Wu , and Z. Zhao , “Heat Stress‐induced Decapping of WUSCHEL mRNA Enhances Stem Cell Thermotolerance in Arabidopsis,” Molecular Plant 17 (2024): 1820–1832, 10.1016/j.molp.2024.10.011. 39468792

[98] C. Wenzl and J. U. Lohmann , “3D imaging Reveals Apical Stem Cell Responses to Ambient Temperature,” Cells and Development 175 (2023): 203850, 10.1016/j.cdev.2023.203850. 37182581

[99] R. Liu , R. Wang , M.‐Z. Lu , and L.‐Q. Wang , “WUSCHEL‐related Homeobox Gene PagWOX11/12a Is Involved in Drought Tolerance through Modulating Reactive Oxygen Species Scavenging in Poplar,” Plant Signaling & Behavior 16 (2021): 1866312, 10.1080/15592324.2020.1866312. 33369514 PMC7889224

[100] J. Lv , Y. Feng , L. Jiang , et al., “Genome‐wide Identification of WOX family Members in Nine Rosaceae Species and a Functional Analysis of MdWOX13‐1 in Drought Resistance,” Plant Science 328 (2023): 111564, 10.1016/j.plantsci.2022.111564. 36549571

[101] T. Laux , K. F. Mayer , J. Berger , and G. Jürgens , “The WUSCHEL Gene Is Required for Shoot and Floral Meristem Integrity in Arabidopsis,” Development 122 (1996): 87–96. 8565856 10.1242/dev.122.1.87

[102] J.‐L. Gallois , C. Woodward , G. V. Reddy , and R. Sablowski , “Combined SHOOT MERISTEMLESS and WUSCHEL Trigger Ectopic Organogenesis in Arabidopsis,” Development 129 (2002): 3207–3217. 12070095 10.1242/dev.129.13.3207

[103] H. Schoof , M. Lenhard , A. Haecker , K. F. Mayer , G. Jürgens , and T. Laux , “The Stem Cell Population of Arabidopsis Shoot Meristems Is Maintained by a Regulatory Loop between the CLAVATA and WUSCHEL Genes,” Cell 100 (2000): 635–644, 10.1016/S0092-8674(00)80700-X. 10761929

[104] U. Brand , J. C. Fletcher , M. Hobe , E. M. Meyerowitz , and R. Simon , “Dependence of Stem Cell Fate in Arabidopsis on a Feedback Loop Regulated by CLV3 Activity,” Science 289 (2000): 617–619, 10.1126/science.289.5479.617. 10915624

[105] T. Suzaki , M. Sato , M. Ashikari , M. Miyoshi , Y. Nagato , and H.‐Y. Hirano , “The Gene FLORAL ORGAN NUMBER1 Regulates Floral Meristem Size in Rice and Encodes a Leucine‐rich Repeat Receptor Kinase Orthologous to Arabidopsis CLAVATA1,” Development 131 (2004): 5649–5657. 15509765 10.1242/dev.01441

[106] L. Liu , J. Gallagher , E. D. Arevalo , et al., “Enhancing Grain‐yield‐related Traits by CRISPR–Cas9 Promoter Editing of Maize CLE Genes,” Nature Plants 7 (2021): 287–294, 10.1038/s41477-021-00858-5. 33619356

[107] C. Pan , X. Wu , K. Markel , et al., “CRISPR–Act3.0 for Highly Efficient Multiplexed Gene Activation in Plants,” Nature Plants 7 (2021): 942–953, 10.1038/s41477-021-00953-7. 34168320

[108] H. Zhou , B. Liu , D. P. Weeks , M. H. Spalding , and B. Yang , “Large Chromosomal Deletions and Heritable Small Genetic Changes Induced by CRISPR/Cas9 in Rice,” Nucleic Acids Research 42 (2014): 10903–10914, 10.1093/nar/gku806. 25200087 PMC4176183

[109] D. Rodríguez‐Leal , Z. H. Lemmon , J. Man , M. E. Bartlett , and Z. B. Lippman , “Engineering Quantitative Trait Variation for Crop Improvement by Genome Editing,” Cell 171 (2017): 470–480. 28919077 10.1016/j.cell.2017.08.030

[110] E. D. Shpak and M. Uzair , “WUSCHEL: the Essential Regulator of the Arabidopsis Shoot Apical Meristem,” Current Opinion in Plant Biology 85 (2025): 102739, 10.1016/j.pbi.2025.102739. 40381531

[111] J. Gregory , X. Liu , Z. Chen , et al., “Transcriptional Corepressors in Maize Maintain Meristem Development,” Plant Physiology 197 (2024): kiae476. 39255069 10.1093/plphys/kiae476 PMC11663565

[112] B. Causier , M. Ashworth , W. Guo , and B. Davies , “The TOPLESS Interactome: a Framework for Gene Repression in Arabidopsis,” Plant Physiology 158 (2012): 423–438, 10.1104/pp.111.186999. 22065421 PMC3252085

[113] T. Li , X. Yang , Y. Yu , et al., “Domestication of Wild Tomato Is Accelerated by Genome Editing,” Nature Biotechnology 36 (2018): 1160–1163, 10.1038/nbt.4273. 30272676

[114] A. Zsögön , T. Čermák , E. R. Naves , et al., “De Novo Domestication of Wild Tomato Using Genome Editing,” Nature Biotechnology 36 (2018): 1211–1216. 10.1038/nbt.4272 30272678

[115] K. Lowe , E. Wu , N. Wang , et al., “Morphogenic Regulators Baby Boom and Wuschel Improve Monocot Transformation,” The Plant Cell 28 (2016): 1998–2015, 10.1105/tpc.16.00124. 27600536 PMC5059793

[116] K. Lowe , M. La Rota , G. Hoerster , et al., “Rapid Genotype “Independent” Zea Mays L. (maize) Transformation via Direct Somatic Embryogenesis,” In Vitro Cellular and Developmental Biology—Plant 54 (2018): 240–252, 10.1007/s11627-018-9905-2. 29780216 PMC5954046

[117] S. Muños , N. Ranc , E. Botton , et al., “Increase in Tomato Locule Number Is Controlled by Two Single‐Nucleotide Polymorphisms Located near WUSCHEL,” Plant Physiology 156 (2011): 2244–2254. 21673133 10.1104/pp.111.173997 PMC3149950

[118] G. Hoerster , N. Wang , L. Ryan , et al., “Use of Non‐integrating Zm‐Wus2 Vectors to Enhance Maize Transformation,” In Vitro Cellular & Developmental Biology—Plant 56 (2020): 265–279, 10.1007/s11627-019-10042-2.

[119] M. Kang , K. Lee , Q. Ji , S. Grosic , and K. Wang , “Enhancing Maize Transformation and Targeted Mutagenesis through the Assistance of Non‐Integrating Wus2 Vector,” Plants 12 (2023): 2799, 10.3390/plants12152799. 37570953 PMC10420852

[120] H. Duan , N. A. Maren , T. G. Ranney , and W. Liu , “New Opportunities for Using WUS/BBM and GRF‐GIF Genes to Enhance Genetic Transformation of Ornamental Plants,” Ornamental Plant Research 2 (2022): 1.

[121] M. F. Maher , R. A. Nasti , M. Vollbrecht , C. G. Starker , M. D. Clark , and D. F. Voytas , “Plant Gene Editing through De Novo Induction of Meristems,” Nature biotechnology 38 (2020): 84–89, 10.1038/s41587-019-0337-2. PMC6954279 31844292

[122] P. Che , E. Wu , M. K. Simon , et al., “Wuschel2 enables Highly Efficient CRISPR/Cas‐targeted Genome Editing during Rapid De Novo Shoot Regeneration in Sorghum,” Communications Biology 5 (2022): 344, 10.1038/s42003-022-03308-w. 35410430 PMC9001672

[123] Z.‐A. Zhang , M.‐Y. Liu , S.‐N. Ren , et al., “Identification of WUSCHEL‐related Homeobox Gene and Truncated Small Peptides in Transformation Efficiency Improvement in Eucalyptus,” BMC Plant Biology 23 (2023): 604, 10.1186/s12870-023-04617-w. 38030990 PMC10688041

[124] Y. Liu , L. Zhang , C. Li , et al., “Establishment of Agrobacterium‐mediated Genetic Transformation and Application of CRISPR/Cas9 Genome‐editing System to Brassica rapa Var. Rapa,” Plant Methods 18 (2022): 98, 10.1186/s13007-022-00931-w. 35933391 PMC9356411

[125] Z. Zhou , Y. Yang , G. Ai , et al., “Boosting Transformation in Wheat by BBM‐WUS,” BioRxiv (2022): 2022, 10.1101/2022.03.13.483388.

