# Supplementary material for: WUSCHEL Transcription Factor: From Stem Cell Maintenance to Crop Improvement
Source: Adv Sci (Weinh). 2026 Mar 31;13(24):e19705. doi: 10.1002/advs.202519705 (PMC13116129; doi:10.1002/advs.202519705)
Supplement: Supplementary file 1 — Supporting Information: advs75044‐sup‐0001‐SuppMat.pdf [file ADVS-13-e19705-s001.pdf]

**Supplementary figure 1.**

The schematic of *WOX* genes structure and homeodomain of WOX proteins. The gene structures of the *WOXs* in *Arabidopsis thaliana, Triticum aestivum L., Bathycoccus prasinos, Brachypodium distachyon, Zea mays, Oryza sativa subsp. Japonica, Phyllostachys edulis, Populus trichocarpa*. Different color represents different domains present in the gene.

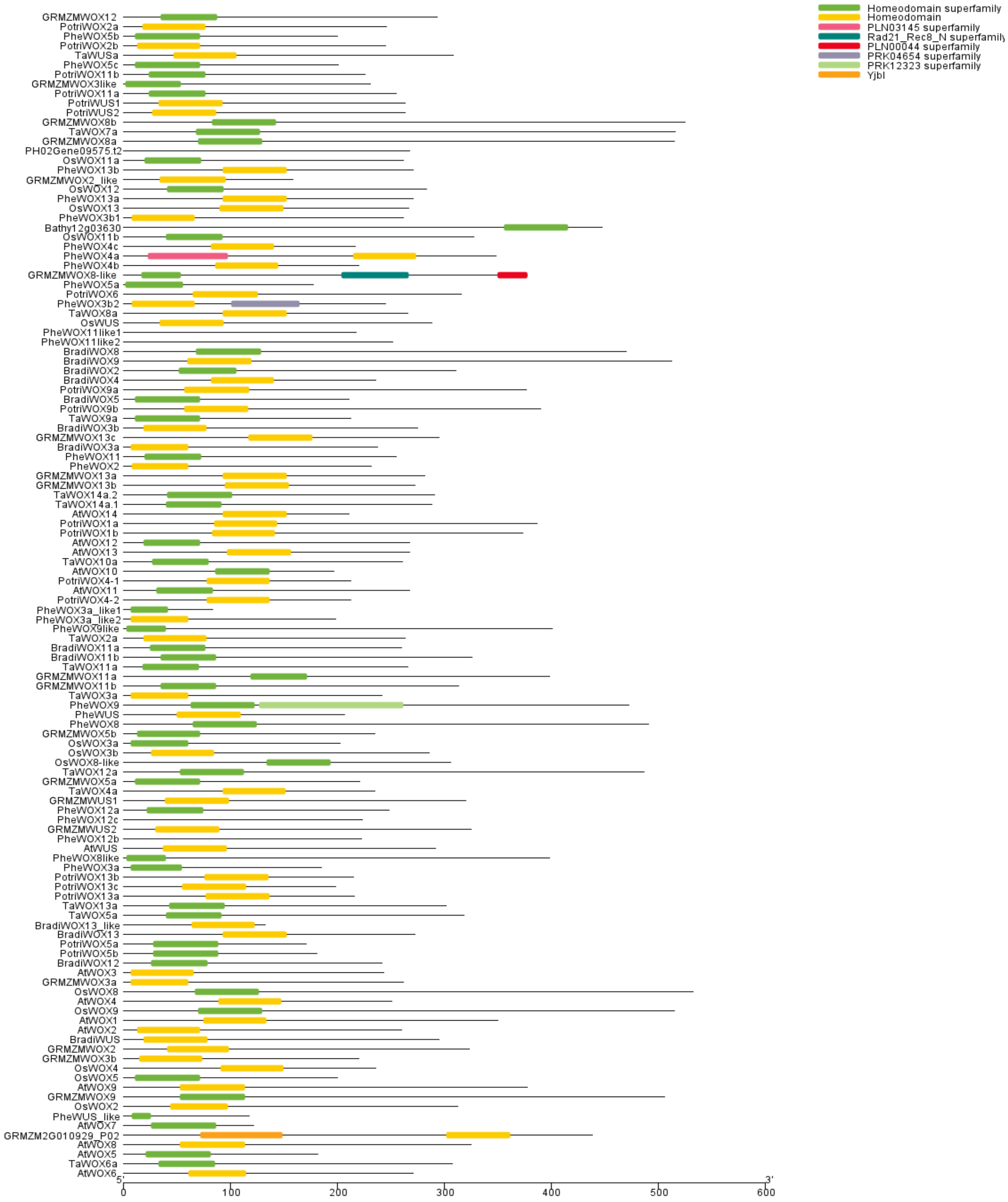

| Sequence name | Weight | Length | Sequence name | Weight | Length |
|---|---|---|---|---|---|
| AtWOX1 | 1.0000 | 350 | AtWOX10 | 1.0000 | 197 |
| AtWOX11 | 1.0000 | 268 | AtWOX12 | 1.0000 | 268 |
| AtWOX13 | 1.0000 | 268 | AtWOX14 | 1.0000 | 211 |
| AtWOX2 | 1.0000 | 260 | AtWOX3 | 1.0000 | 244 |
| AtWOX4 | 1.0000 | 251 | AtWOX5 | 1.0000 | 182 |
| AtWOX6 | 1.0000 | 271 | AtWOX7 | 1.0000 | 122 |
| AtWOX8 | 1.0000 | 325 | AtWOX9 | 1.0000 | 378 |
| AtWUS | 1.0000 | 292 | TaWOX2a | 1.0000 | 264 |
| TaWOX12a | 1.0000 | 487 | TaWOX11a | 1.0000 | 266 |
| TaWOX4a | 1.0000 | 235 | TaWUSa | 1.0000 | 309 |
| TaWOX14a.2 | 1.0000 | 291 | TaWOX9a | 1.0000 | 213 |
| TaWOX14a.1 | 1.0000 | 289 | TaWOX7a | 1.0000 | 516 |
| TaWOX10a | 1.0000 | 261 | TaWOX13a | 1.0000 | 302 |
| TaWOX8a | 1.0000 | 266 | TaWOX6a | 1.0000 | 308 |
| TaWOX3a | 1.0000 | 242 | TaWOX5a | 1.0000 | 319 |
| Bathy12g03630 | 1.0000 | 448 | BradiWOX11a | 1.0000 | 260 |
| BradiWOX11b | 1.0000 | 326 | BradiWOX12 | 1.0000 | 242 |
| BradiWOX13 | 1.0000 | 273 | BradiWOX13_like | 1.0000 | 133 |
| BradiWOX2 | 1.0000 | 311 | BradiWOX3a | 1.0000 | 238 |
| BradiWOX3b | 1.0000 | 275 | BradiWOX4 | 1.0000 | 236 |
| BradiWOX5 | 1.0000 | 211 | BradiWOX9 | 1.0000 | 513 |
| BradiWOX8 | 1.0000 | 470 | BradiWUS | 1.0000 | 295 |
| GRMZM2G010929_P02 | 1.0000 | 439 | GRMZMWOX11a | 1.0000 | 399 |
| GRMZMWOX11b | 1.0000 | 314 | GRMZMWOX12 | 1.0000 | 294 |
| GRMZMWOX13a | 1.0000 | 282 | GRMZMWOX13b | 1.0000 | 273 |
| GRMZMWOX13c | 1.0000 | 295 | GRMZMWOX2 | 1.0000 | 324 |
| GRMZMWOX2_like | 1.0000 | 159 | GRMZMWOX3a | 1.0000 | 262 |
| GRMZMWOX3b | 1.0000 | 220 | GRMZMWOX3like | 1.0000 | 231 |
| GRMZMWOX5a | 1.0000 | 221 | GRMZMWOX5b | 1.0000 | 235 |
| GRMZMWOX8-like | 1.0000 | 378 | GRMZMWOX8a | 1.0000 | 515 |
| GRMZMWOX8b | 1.0000 | 525 | GRMZMWOX9 | 1.0000 | 506 |

| | | | | | |
|---|---|---|---|---|---|
| GRMZMWUS1 | 1.0000 | 320 | GRMZMWUS2 | 1.0000 | 325 |
| OsWOX11a | 1.0000 | 262 | OsWOX11b | 1.0000 | 328 |
| OsWOX12 | 1.0000 | 284 | OsWOX13 | 1.0000 | 267 |
| OsWOX2 | 1.0000 | 313 | OsWOX3a | 1.0000 | 203 |
| OsWOX3b | 1.0000 | 286 | OsWOX4 | 1.0000 | 236 |
| OsWOX5 | 1.0000 | 200 | OsWOX8 | 1.0000 | 533 |
| OsWOX8-like | 1.0000 | 306 | OsWOX9 | 1.0000 | 515 |
| OsWUS | 1.0000 | 289 | PH02Gene09575.t2 | 1.0000 | 268 |
| PheWOX11like2 | 1.0000 | 252 | PheWOX11 | 1.0000 | 255 |
| PheWOX11like1 | 1.0000 | 218 | PheWOX12a | 1.0000 | 249 |
| PheWOX12b | 1.0000 | 223 | PheWOX12c | 1.0000 | 224 |
| PheWOX13a | 1.0000 | 271 | PheWOX13b | 1.0000 | 271 |
| PheWOX2 | 1.0000 | 232 | PheWOX3a | 1.0000 | 185 |
| PheWOX3a_like1 | 1.0000 | 84 | PheWOX3a_like2 | 1.0000 | 199 |
| PheWOX3b1 | 1.0000 | 262 | PheWOX3b2 | 1.0000 | 245 |
| PheWOX4a | 1.0000 | 349 | PheWOX4b | 1.0000 | 220 |
| PheWOX4c | 1.0000 | 217 | PheWOX5a | 1.0000 | 178 |
| PheWOX5b | 1.0000 | 200 | PheWOX5c | 1.0000 | 201 |
| PheWOX8 | 1.0000 | 491 | PheWOX8like | 1.0000 | 399 |
| PheWOX9like | 1.0000 | 401 | PheWOX9 | 1.0000 | 473 |
| PheWUS | 1.0000 | 207 | PheWUS_like | 1.0000 | 118 |
| PotriWOX11a | 1.0000 | 255 | PotriWOX11b | 1.0000 | 226 |
| PotriWOX13c | 1.0000 | 199 | PotriWOX13a | 1.0000 | 216 |
| PotriWOX13b | 1.0000 | 215 | PotriWOX1a | 1.0000 | 387 |
| PotriWOX1b | 1.0000 | 374 | PotriWOX2a | 1.0000 | 246 |
| PotriWOX2b | 1.0000 | 245 | PotriWOX4-2 | 1.0000 | 213 |
| PotriWOX4-1 | 1.0000 | 213 | PotriWOX5a | 1.0000 | 171 |
| PotriWOX5b | 1.0000 | 181 | PotriWOX6 | 1.0000 | 316 |
| PotriWOX9a | 1.0000 | 377 | PotriWOX9b | 1.0000 | 390 |
| PotriWUS1 | 1.0000 | 264 | PotriWUS2 | 1.0000 | 264 |

**Supplementary figure 2. Motif domain and location.**

The conserved motifs among the members are highlighted in colored boxes with an arranged number, and the sequences of the motifs are listed in Table. Colors of boxes indicate different motif numbers; length of box shows motif length. 10 Conserved motifs in WOX proteins were identified using the MEME suite program and visualized by TBtools.

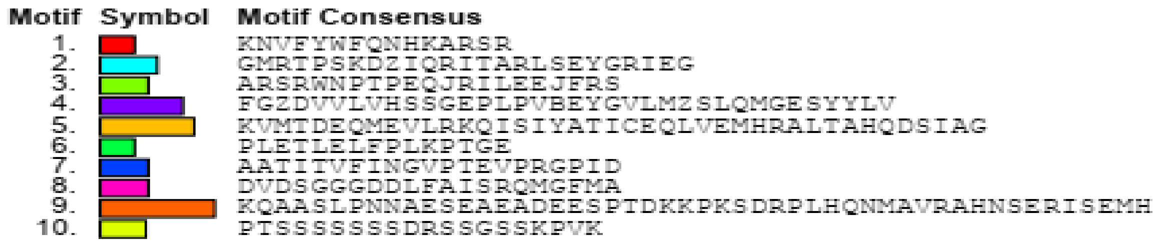


**Table**

| Motif | Symbol | Motif Consensus |
|---|---|---|
| 1. | | KNVFYWFQNHKARSR |
| 2. | | GMRTPSKDZIQRITARLSEYGRIEG |
| 3. | | ARSRWNPTPEQJRILEEJFRS |
| 4. | | FGZDVVLVHSSGEPLPVBEYGVLMZSLQMGESYYLV |
| 5. | | KVMTDEQMEVLRKQISIYATICEQLVEMHRALTAHQDSIAG |
| 6. | | PLETLELFPLKPTGE |
| 7. | | AATITVFINGVPTEVPRGPID |
| 8. | | DVDSGGGDDLFAISRQMGFMA |
| 9. | KQAASLPNNAESEAEADEESPTDKKPKSDRPLHQNMAVRAHNSERISEMH | |
| 10. | | PTSSSSSSSDRSSGSSKPVK |

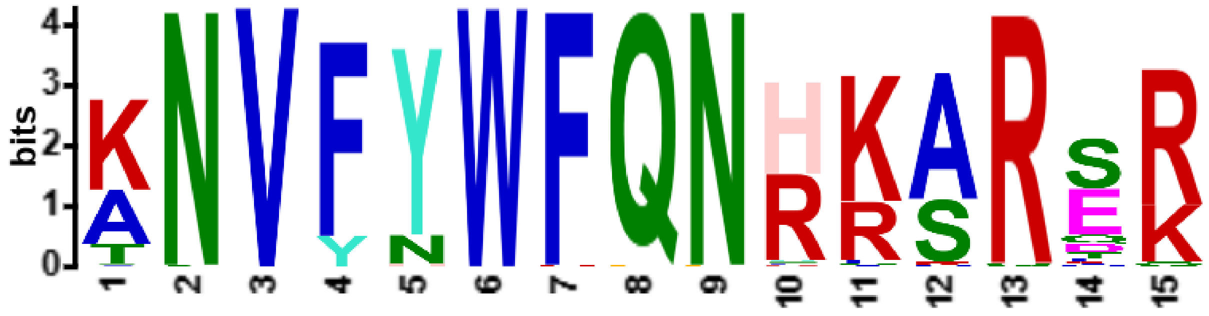


2

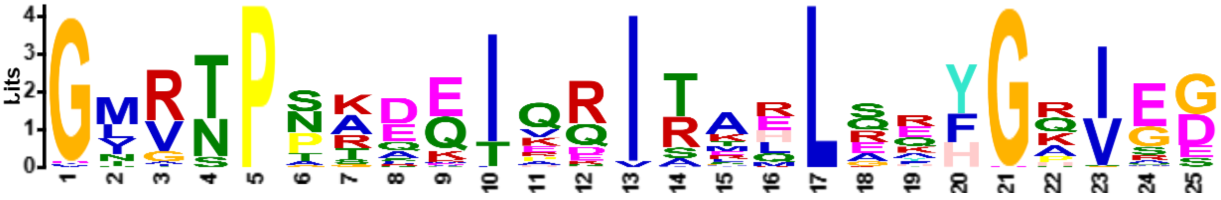


3

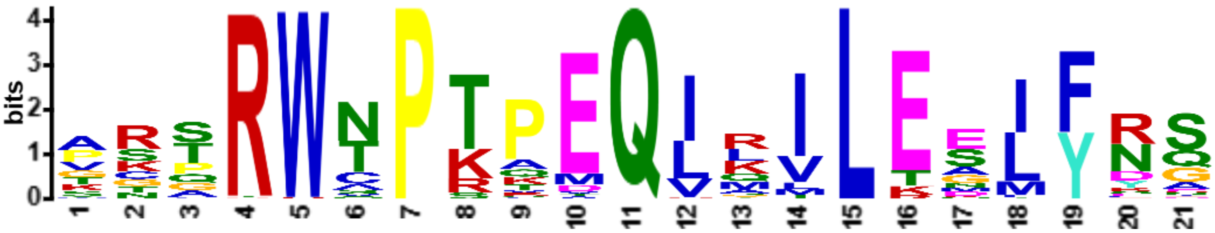

4

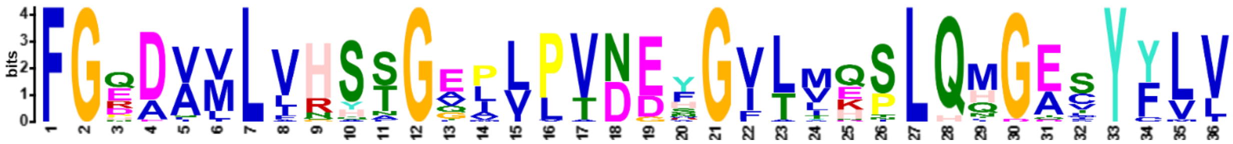

5

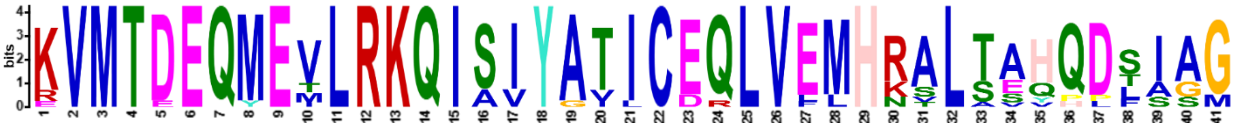

6

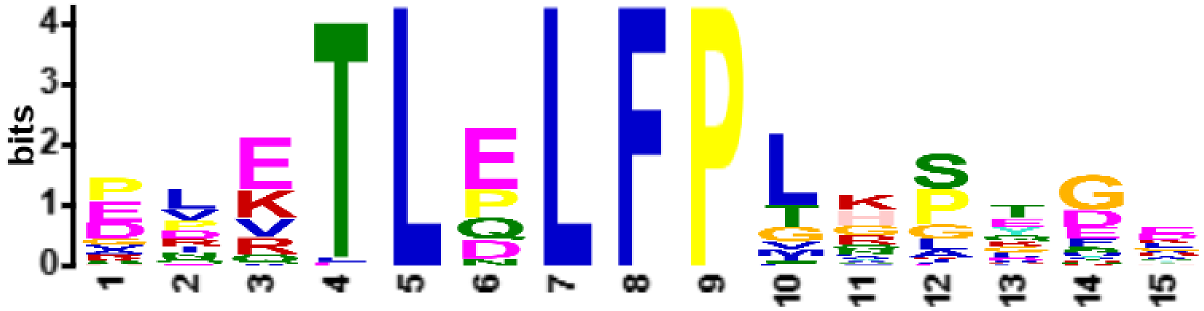

7

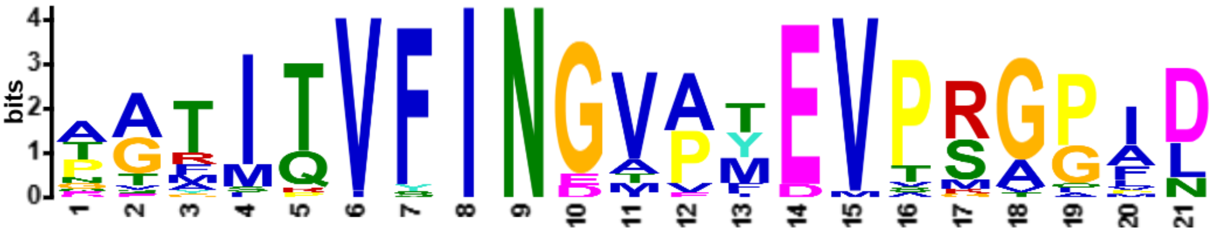

8

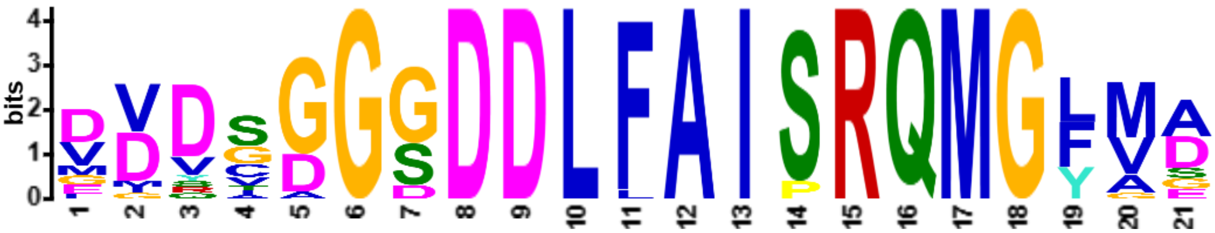

9

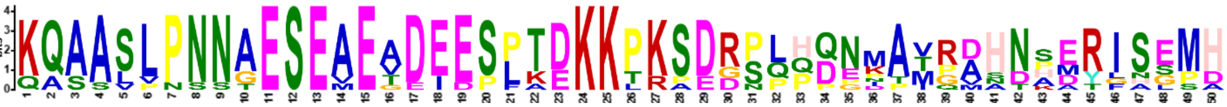

10

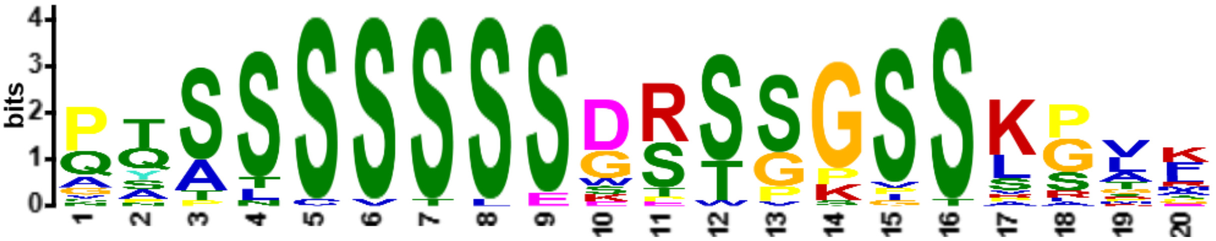

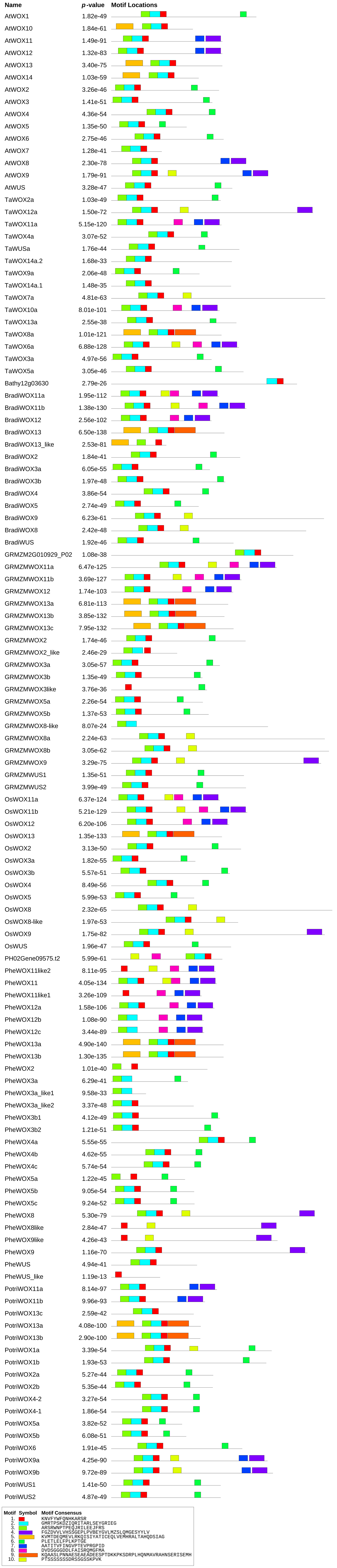
Name
p-value
Motif Locations
AtWOX1 1.82e-49
AtWOX10 1.84e-61
AtWOX11 1.49e-91
AtWOX12 1.32e-83
AtWOX13 3.40e-75
AtWOX14 1.03e-59
AtWOX2 3.26e-46
AtWOX3 1.41e-51
AtWOX4 4.36e-54
AtWOX5 1.35e-50
AtWOX6 2.75e-46
AtWOX7 1.28e-41
AtWOX8 2.30e-78
AtWOX9 1.79e-91
AtWUS 3.28e-47
TaWOX2a 1.03e-49
TaWOX12a 1.50e-72
TaWOX11a 5.15e-120
TaWOX4a 3.07e-52
TaWUSa 1.76e-44
TaWOX14a.2 1.68e-33
TaWOX9a 2.06e-48
TaWOX14a.1 1.48e-35
TaWOX7a 4.81e-63
TaWOX10a 8.01e-101
TaWOX13a 2.55e-38
TaWOX8a 1.01e-121
TaWOX6a 6.88e-128
TaWOX3a 4.97e-56
TaWOX5a 3.05e-46
Bathy12g03630 2.79e-26
BradiWOX11a 1.95e-112
BradiWOX11b 1.38e-130
BradiWOX12 2.56e-102
BradiWOX13 6.50e-138
BradiWOX13_like 2.53e-81
BradiWOX2 1.84e-41
BradiWOX3a 6.05e-55
BradiWOX3b 1.97e-48
BradiWOX4 3.86e-54
BradiWOX5 2.74e-49
BradiWOX9 6.23e-61
BradiWOX8 2.42e-48
BradiWUS 1.92e-46
GRMZM2G010929_P02 1.08e-38
GRMZMWOX11a 6.47e-125
GRMZMWOX11b 3.69e-127
GRMZMWOX12 1.74e-103
GRMZMWOX13a 6.81e-113
GRMZMWOX13b 3.85e-132
GRMZMWOX13c 7.95e-132
GRMZMWOX2 1.74e-46
GRMZMWOX2_like 2.46e-29
GRMZMWOX3a 3.05e-57
GRMZMWOX3b 1.35e-49
GRMZMWOX3like 3.76e-36
GRMZMWOX5a 2.26e-54
GRMZMWOX5b 1.37e-53
GRMZMWOX8-like 8.07e-24
GRMZMWOX8a 2.24e-63
GRMZMWOX8b 3.05e-62
GRMZMWOX9 3.29e-75
GRMZMWUS1 1.35e-51
GRMZMWUS2 3.99e-49
OsWOX11a 6.37e-124
OsWOX11b 5.21e-129
OsWOX12 6.20e-106
OsWOX13 1.35e-133
OsWOX2 3.13e-50
OsWOX3a 1.82e-55
OsWOX3b 5.57e-51
OsWOX4 8.49e-56
OsWOX5 5.99e-53
OsWOX8 2.32e-65
OsWOX8-like 1.97e-53
OsWOX9 1.75e-82
OsWUS 1.96e-47
PH02Gene09575.t2 5.99e-61
PheWOX11like2 8.11e-95
PheWOX11 4.05e-134
PheWOX11like1 3.26e-109
PheWOX12a 1.58e-106
PheWOX12b 1.08e-90
PheWOX12c 3.44e-89
PheWOX13a 4.90e-140
PheWOX13b 1.30e-135
PheWOX2 1.01e-40
PheWOX3a 6.29e-41
PheWOX3a_like1 9.58e-33
PheWOX3a_like2 3.37e-48
PheWOX3b1 4.12e-49
PheWOX3b2 1.21e-51
PheWOX4a 5.55e-55
PheWOX4b 4.62e-55
PheWOX4c 5.74e-54
PheWOX5a 1.22e-45
PheWOX5b 9.05e-54
PheWOX5c 9.24e-52
PheWOX8 5.30e-79
PheWOX8like 2.84e-47
PheWOX9like 4.26e-43
PheWOX9 1.16e-70
PheWUS 4.94e-41
PheWUS_like 1.19e-13
PotriWOX11a 8.14e-97
PotriWOX11b 9.96e-93
PotriWOX13c 2.59e-42
PotriWOX13a 4.08e-100
PotriWOX13b 2.90e-100
PotriWOX1a 3.39e-54
PotriWOX1b 1.93e-53
PotriWOX2a 5.27e-44
PotriWOX2b 5.35e-44
PotriWOX4-2 3.27e-54
PotriWOX4-1 1.86e-54
PotriWOX5a 3.82e-52
PotriWOX5b 6.08e-51
PotriWOX6 1.91e-45
PotriWOX9a 4.25e-90
PotriWOX9b 9.72e-89
PotriWUS1 1.41e-50
PotriWUS2 4.87e-49
Motif Symbol Motif Consensus
1. KNVFYWFQNHKARSR
2. GMRTPSKDZIQRITARLSEYGRIEG
3. ARSRWNPTPEQJRILEEJFRS
4. FGZDVVLVHSSGEPLPVBEYGVLMZSLQMGESYYLV
5. KVMTDEQMEVLRKQISIYATICEQLVEMHRALTAHQDSIAG
6. PLETLELFPLKPTGE
7. AATITVFINGVPTEVPRGPID
8. DVDSGGGDDLFAISRQMGFMA
9. KQAASLPNNAESEAEADEESPTDKKPKSDRPLHQNMAVRAHNSERISEMH
10. PTSSSSSSSDRSSGSSKPVK
